# A lignan induces lysosomal dependent degradation of FoxM1 protein to suppress β-catenin nuclear translocation

**DOI:** 10.1038/srep45951

**Published:** 2017-04-05

**Authors:** Guang-zhi Dong, Ji Hye Jeong, Yu-ih Lee, Yeong Eun Han, Jung Sook Shin, Yoon-Jung Kim, Raok Jeon, Young Hwa Kim, Tae Jun Park, Keun Il Kim, Jae-Ha Ryu

**Affiliations:** 1 Research Center for Cell Fate Control and College of Pharmacy, Sookmyung Women’s University, Seoul 04310, Korea; 2 Department of Biochemistry and Molecular Biology, School of Medicine, Ajou University, Suwon, 16499, Korea; 3 Department of Biological Science, Sookmyung Women’s University, Seoul 04310, Korea

## Abstract

Colon cancer is one of the most common cancers. In this study, we isolated a lignan [(−)-(2R,3R)-1,4-O-diferuloylsecoisolariciresinol, DFS] from *Alnus japonica* (Betulaceae) and investigated its biological activity and mechanism of action on colon cancer. DFS reduced the viability of colon cancer cells and induced cell cycle arrest. DFS also suppressed β-catenin nuclear translocation and β-catenin target gene expression through a reduction in FoxM1 protein. To assess the mechanism of the action of DFS, we investigated the effect of DFS on endogenous and exogenous FoxM1 protein degradation in colon cancer cells. DFS-induced FoxM1 protein degradation was suppressed by lysosomal inhibitors, chloroquine and bafilomycin A1, but not by knock-down of proteasomal proteins. The mechanism of DFS for FoxM1 degradation is lysosomal dependent, which was not reported before. Furthermore, we found that FoxM1 degradation was partially lysosomal-dependent under normal conditions. These observations indicate that DFS from *A. japonica* suppresses colon cancer cell proliferation by reducing β-catenin nuclear translocation. DFS induces lysosomal-dependent FoxM1 protein degradation. This is the first report on the lysosomal degradation of FoxM1 by a small molecule. DFS may be useful in treating cancers that feature the elevated expression of FoxM1.

The Wnt/β-catenin signaling pathway plays a primary role in cellular differentiation and proliferation. Beta-catenin forms a complex with APC/Axin/GSK3β and is degraded by the proteasome under Wnt-free conditions. However, the Wnt/β-catenin pathway is constitutively activated in most sporadic and hereditary colorectal tumors caused by mutations in Wnt/β-catenin pathway-related molecules, such as adenomatous polyposis coli (APC) and β-catenin^1^. Aberrantly activated β-catenin increases nuclear translocation of other oncogenes^2^,^3^ and binds to T-cell factor/lymphoid enhancer factor transcription factors to promote expression of target genes, such as cyclin D1, survivin, and c-Myc, which play key roles in cellular differentiation and proliferation^4^,^5^. Thus, aberrantly activated Wnt/β-catenin signaling is regarded as a target for the chemoprevention and treatment of colorectal cancer.

FoxM1 is a member of the Forkhead box transcription factor family. The varied biological activities of FoxM1, include regulation of cellular proliferation, DNA damage repair, angiogenesis, apoptosis, and tumorigenesis^6^. From the early stage of tumor development to later metastasis, FoxM1 expression is highly elevated in a variety of cancers^6^,^7^. Elevation in FoxM1 levels promotes cancer initiation and maintenance through regulation of the progression of cancer cell cycle and proliferation^6^,^7^. For example, elevation in FoxM1 levels promotes development and proliferation of colon adenocarcinomas *in vivo,* and depletion of FoxM1 reduces colon cancer cell growth *in vitro*^8^. Furthermore, FoxM1 also increases the transcriptional activity of other oncogenes to promote tumorigenesis. For example, FoxM1 interacts with β-catenin and promotes the nuclear translocation of β-catenin to promote glioma stem cell self-renewal and tumorigenesis through increased expression of β-catenin target genes^3^. Therefore, targeting FoxM1 is a good strategy for chemoprevention and treatment of colorectal cancer.

*Alnus japonica* (Betulaceae) grows in the low mountainous areas of Korea, northeast China, and Japan. It has been used in traditional oriental medicine to treat fever, hemorrhage, diarrhea, and alcoholism. Recent studies have shown that *Alnus japonica* has various phytochemicals, such as diarylheptanoids, triterpenoids, and flavonoids^9^,^10^,^11^,^12^,^13^,^14^. In this study, we isolated a lignan [(−)-(2R,3R)-1,4-O-diferuloylsecoisolariciresinol, DFS] from *Alnus japonica* and explored its activity against colon cancer. DFS was first reported by Nomura and Tokoroyama^15^ and its cytotoxic action against several cancer cell types has been described^16^,^17^,^18^. Presently, we describe the ability of DFS to block β-catenin nuclear translocation through the lysosomal-dependent degradation of FoxM1 protein.

## Results

### DFS suppresses the β-catenin pathway

**Figure 1:**
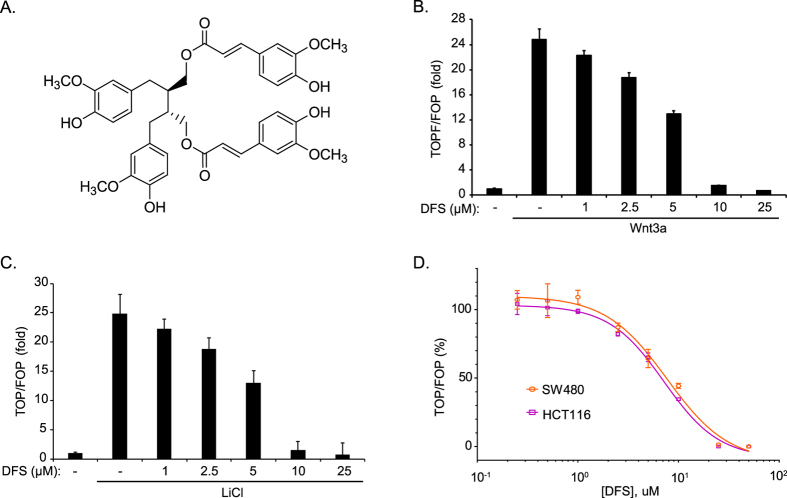
DFS suppresses the β-catenin signaling pathway. The structure of DFS (**A**). TOPFlash or FOPFlash reporter expressed HEK293 cells were treated with the indicated concentrations of DFS in the presence of Wnt3a (**B**) or LiCl (**C**) for 16 h and TOPFlash activity was measured. SW480 and HCT116 colon cancer cells were transiently transfected with the TOPFlash plasmid and treated with the indicated concentrations of DFS for 16 h, TOPFlash activity was measured (**D**).

TOPFlash and FOPFlash reporter cell lines were used to test the effects of DFS (Fig. 1A) on the Wnt/β-catenin pathway. Treatment with Wnt3a-conditioned media (CM) significantly increased TOPFlash activity, and treatment with DFS suppressed Wnt3a-induced TOPFlash activity in a dose-dependent manner (Fig. 1B). To test whether GSK-3β is involved in the inhibition of β-catenin transcription, we treated HEK293 cells with LiCl as an inhibitor of GSK-3β^14^. DFS suppressed LiCl-induced TOPFlash activity in a dose-dependent manner (Fig. 1C). These data indicate that DFS suppresses the β-catenin pathway in a GSK-3β-independent manner.

Next, we tested the ability of DFS to suppress the Wnt/β-catenin pathway in colon cancer cells. SW480 and HCT116 cells (adenomatous polyposis [APC] mutated or β-catenin mutated, respectively) were transiently transfected with the TOPFlash plasmid and treated with DFS to assess luciferase activity. DFS significantly suppressed TOPFlash activity in both colon cancer cell types with an IC_50_ value of 7.68 μM and 7.17 μM, respectively (Fig. 1D). These data indicate that DFS suppresses the β-catenin pathway in both APC mutated and β-catenin mutated colon cancer cells.

### DFS suppresses colon cancer cell proliferation and induces cell death

**Figure 2:**
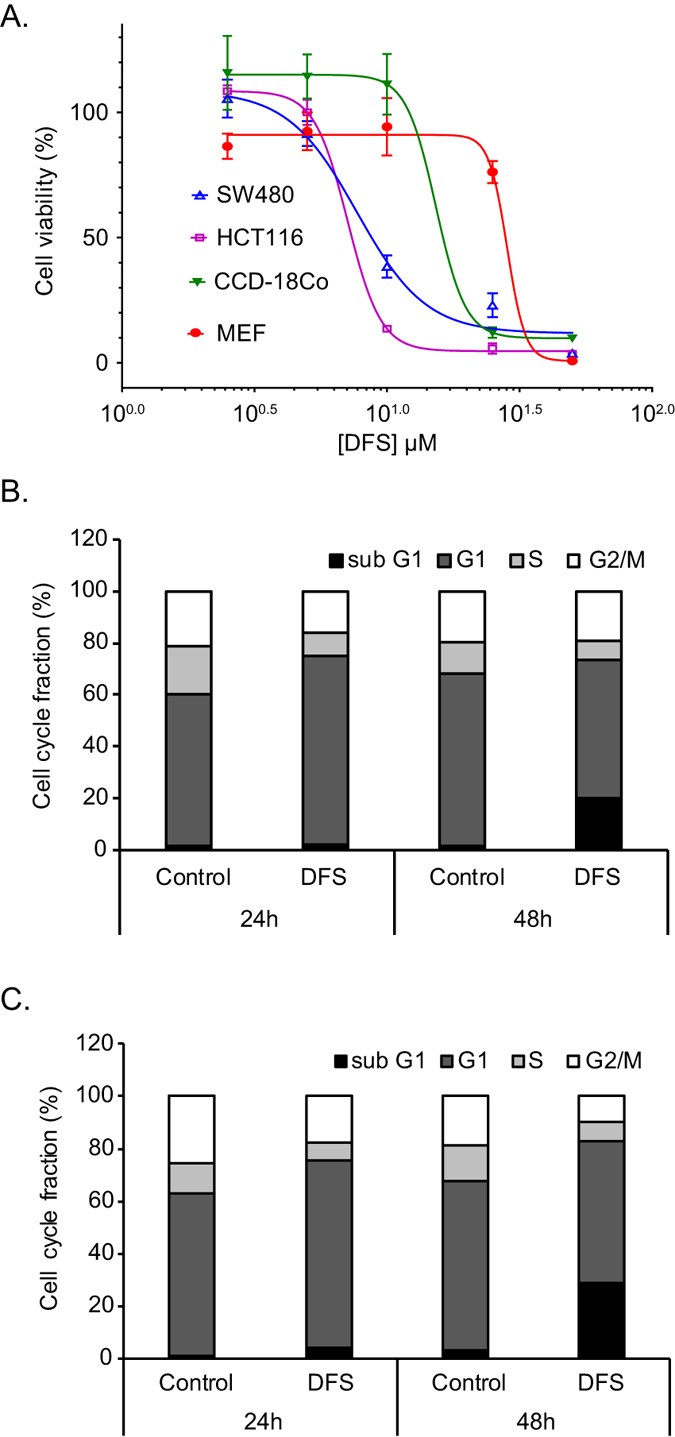
DFS suppresses colon cancer cell proliferation and induces cell death. Cell viability was determined with the MTT assay after treatment with indicated concentration of DFS for 48 h (**A**). SW480 and HCT116 colon cancer cell cycle was analyzed by FACS after treatment with 10 μM DFS for the indicated times (**B,C**).

As abnormal activation of the Wnt/β-catenin pathway is the main cause of colon cancer cell proliferation^19^, we tested the inhibitory potential of DFS on the growth of colon cancer cells using the MTT and cell cycle assays. DFS reduced cell viability of SW480 and HCT116 (Fig. 2A) colon cancer cells in a dose-dependent manner. In the case of normal cells, such as colon CCD-18Co and mouse embryonic fibroblast (MEF), DFS displayed only marginal cytotoxicity at a high dose of 25 μM (Fig. 2A). Cell cycle analysis showed that DFS significantly induced G_1_ phase arrest in both SW480 and HCT116 colon cancer cells at 24 h, and induced cell death at 48 h (Fig. 2B,C). Taken together, the data indicate that DFS has anti-proliferative activity against aberrantly activated β-catenin-induced colon cancer cell and decreases the survival of SW480 and HCT116 colon cancer cells.

### DFS suppresses nuclear location of β-catenin by decreasing the level of FoxM1 protein

**Figure 3:**
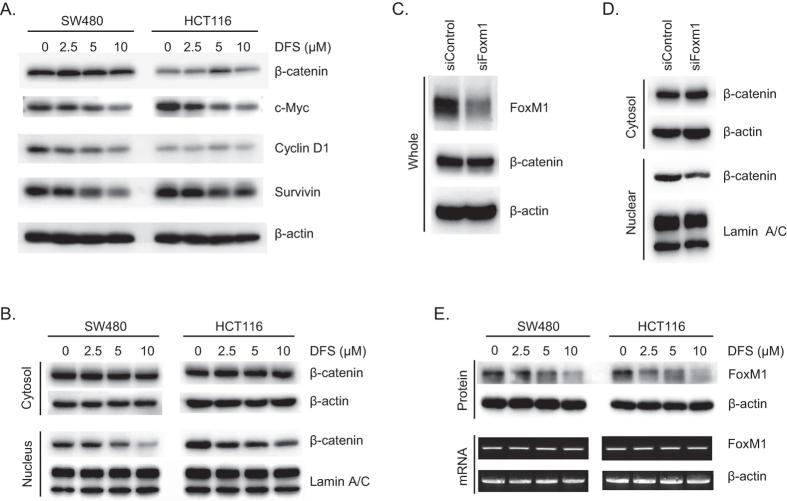
DFS suppresses β-catenin nuclear translocation by reducing the level of FoxM1 protein. SW480 and HCT116 colon cancer cells were treated with the indicated concentrations of DFS for 16 h and the protein levels of β-catenin and its target genes were measured (**A**). The cytosolic and nuclear protein levels of β-catenin were determined by Western blot (**B**). SW480 cells were transfected with siRNA against FoxM1 and the localization of β-catenin was determined by Western blot (**C,D**). SW480 and HCT116 colon cancer cells were treated with the indicated concentrations of DFS for 16 h, and the protein and mRNA levels of FoxM1 were determined by Western blot and RT-PCR (**E**).

To confirm the inhibitory effects of DFS on the Wnt/β-catenin pathway, β-catenin levels and levels of its target genes were determined by Western blotting. DFS siginficantly reduced the levels of β-catenin target genes, such as cyclin D1, survivin, and c-Myc in SW480 and HCT116 cells without altering the total level of β-catenin (Fig. 3A). Interestingly, the nuclear level of β-catenin was decreased by DFS in a dose-dependent manner in colon cancer cells, whereas the cytosolic level was not changed (Fig. 3B). These data indicate that DFS suppresses the Wnt/β-catenin pathway by blocking nuclear translocation of β-catenin in colon cancer cells. As FoxM1 modulates β-catenin nuclear translocation^3^, we tested the effect of DFS on FoxM1 levels in colon cancer cells. Knock-down of FoxM1 decreased nuclear translocation of β-catenin in SW480 cells (Fig. 3C,D). FoxM1 protein levels were reduced by DFS treatment, while the mRNA level of FoxM1 was not affected in either SW480 or HCT116 cells (Fig. 3E). These data indicate that DFS inhibits the nuclear translocation of β-catenin in colon cancer cells by modulating the level of FoxM1 protein.

### DFS destabilizes FoxM1 protein in colon cancer cells

**Figure 4:**
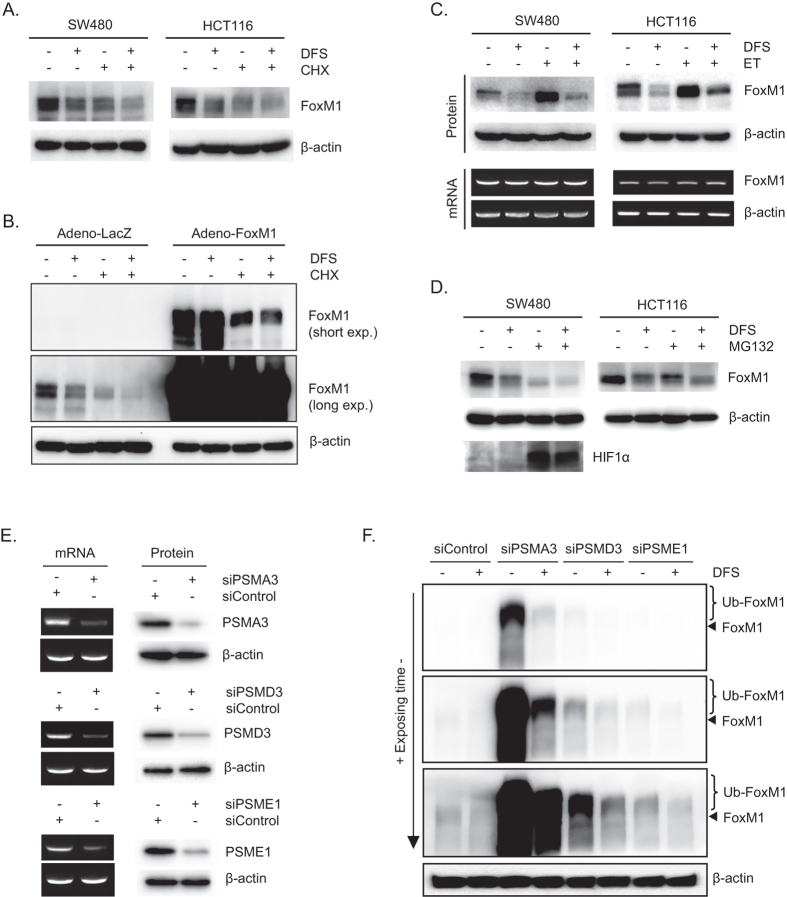
DFS induces lysosomal-dependent FoxM1 protein degradation. SW480 and HCT116 colon cancer cells were treated with DFS and cycloheximide (CHX) for 14 h and the protein levels of FoxM1 were determined by Western blot (**A**). HCT116 colon cancer cells were infected with adeno-FoxM1 virus and treated with DFS and CHX for 14 h, then the protein levels of FoxM1 were determined by Western blot (**B**). SW480 and HCT116 colon cancer cells were treated with DFS and Etoposide (ET) for 16 h and the protein and mRNA levels of FoxM1 were determined by Western blot and RT-PCR (**C**). SW480 and HCT116 colon cancer cells were treated with DFS and MG132 for 16 h and the protein and mRNA levels of FoxM1 were determined by Western blot and RT-PCR. Then, the membrane was reprobed by stripping buffer and HIF1α protein levels were measured to assess MG132 activity (**D**). SW480 cells were transfected with the indicated siRNAs and related gene expressions were determined by RT-PCR and Western blot assay (**E**). SiRNA-transfected SW480 cells were treated with DFS for 16 h and FoxM1 protein levels were determined by Western blot (**F**). Black triangle denotes original FoxM1 and the brace denotes post-modified FoxM1.

Cycloheximide was used to investigate the mechanism of action of DFS involved in suppressing FoxM1 levels. Treatment with DFS reduced FoxM1 levels in cycloheximide-treated and -untreated SW480 and HCT116 cells (Fig. 4A). Furthermore, DFS induced degradation of endogenously expressed FoxM1 in control (LacZ) adenovirus-infected HCT116 cells (Fig. 4B, left panel). DFS also induced degradation of exogenously expressed FoxM1 in cycloheximide-treaded HCT116 cells (Fig. 4B, right panel). These data indicate that DFS destabilizes the FoxM1 protein, which suppresses β-catenin nuclear translocation.

To confirm the effects of DFS on FoxM1 protein degradation, we used etoposide, a clinically available anti-cancer drug that increases FoxM1 by stabilizing FoxM1 protein^20^,^21^. Etoposide increased FoxM1 levels without changing mRNA levels (Fig. 4C). However, treatment with DFS reduced protein levels of etoposide-induced FoxM1 in both SW480 and HCT116 cells (Fig. 4C). Thus, DFS diminishes the stability of FoxM1 protein, which suppresses β-catenin nuclear translocation.

### DFS induces lysosomal-dependent degradation of FoxM1 protein

Protein degradation is dependent on the proteasome^22^. To investigate the mechanism of action of DFS for FoxM1 protein degradation, colon cancer cells were treated with the MG132 proteasome inhibitor. Treatment with MG132 reduced FoxM1 levels in both SW480 and HCT116 cells (Fig. 4D). MG132 inhibits FoxM1 transcriptional activity and FoxM1 expression in several cancer cell types^23^. Presently, co-treatment with DFS and MG132 further reduced FoxM1 levels than treatment with either DFS or MG132 alone (Fig. 4D). Proteasomal inhibitory activity of MG132 was confirmed by measuring the level of hypoxia inducible factor 1α (HIF1α) in SW480 cells (Fig. 4D).

To further investigate whether the proteasomal pathway is involved in DFS-induced FoxM1 degradation, siRNAs were used (Fig. 4E). Knock-down of PSMA3 (20S proteasomal protein), PSMD3 (19S proteasomal protein), and PSME1 (11S proteasomal protein) resulted in increased size and quantity of FoxM1 compared with control colon cancer cells (Fig. 4F, brace). These observations and knowledge that FoxM1 can be ubiquitinated and degraded by proteasome^20^,^21^ indicate that knock-down of proteasomal proteins suppresses FoxM1 protein degradation, and that the 20S, 19S, and 11S proteasomes are involved in FoxM1 protein degradation. However, DFS induced FoxM1 degradation in proteasomal protein knock-down cells (Fig. 4F). These data indicate that other mechanism than proteasomal degradataion might be involved in DFS-induced FoxM1 degradation.

**Figure 5:**
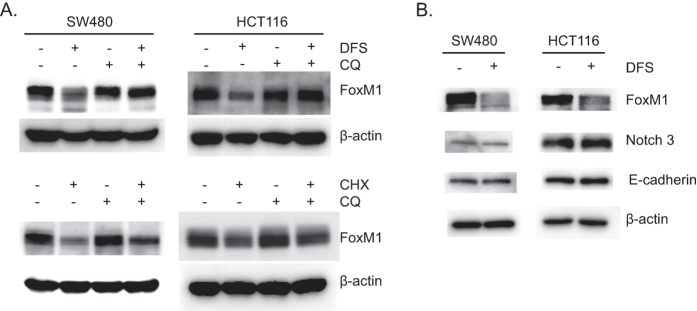
DFS induces lysosomal-dependent FoxM1 protein degradation. Protein levels of FoxM1 were determined by Western blot in SW480 and HCT116 cells after treatment with DFS and chloroquine (CQ) or cycloheximide (CHX) (**A**). Colon cancer cells were treated with DFS and the levels of E-cadherin and Notch3 were determined by Western blot assay (**B**).

To assess whether the lysosomal degradation pathway is involved in DFS-induced FoxM1 degradation, the lysosomal inhibitors, chloroquine and bafilomycin A1, were used. DFS-induced FoxM1 degradation was blocked by treatment with chloroquine in colon cancer cells (Fig. 5A), while the levels of Notch 3 and E-cadherin were not changed by DFS (Fig. 5B). Notch 3 and E-cadherin were also known to be targeted for lysosomal degradation^24^,^25^. These data indicate that DFS induced FoxM1 degradation in a lysosomal-dependent manner. Treatment with chloroquine slightly restored FoxM1 levels in cycloheximide-treated colon cancer cells (Fig. 5A), which means that FoxM1 degradation is partially dependent on the lysosome under normal conditions. Similar results were observed when bafilomycin A1, another lysosomal degradation inhibitor was treated SW480 colon cancer cells (Fig. S8).

### DFS has antitumor activity in a tumor xenograft mouse model

**Figure 6:**
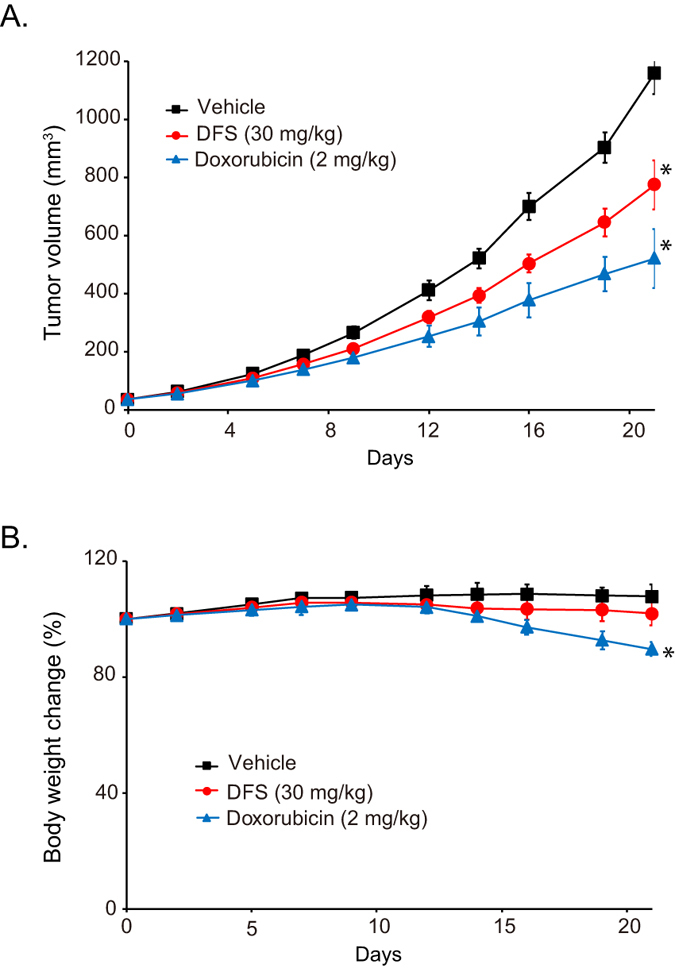
DFS inhibits the growth of tumor *in vivo*. SW480 cells (9 × 10^6^ cells/mouse) were inoculated subcutaneously into the right flank of nude mice (n = 5). Treatment was initiated when tumor volumes reached approximately ~40 mm^3^. DFS (30 mg/kg body weight) was administered intraperitoneally 5 days a week for 21 days. On day 21, mice were sacrificed and tumor volume was measured (**A**). The asterisk (*) indicated significancy compared to vehicle group (*p* < 0.01). Body weight was measured during the experiments as an indication of toxicity (**B**).

The antitumor activity of DFS was evaluated in a nude mouse xenograft model with implanted SW480 human colon cancer cells. Mice were treated with DFS 5 days a week by intraperitoneal injection. The tumor volumes in DFS-administered groups with 30 mg/kg were significantly reduced (*p <  0.01) compared with the vehicle at the termination of the experiment on day 21 (Fig. 6A). No overt toxicity or change in body weight was shown in the treatment groups compared to the vehicle-treated control group (Fig. 6B). These results confirm that DFS effectively inhibits the tumor growth of human colorectal cancer cells *in vivo*.

## Discussion

Colorectal cancer is a common cancer that is responsible for approximately 10% of all cancer-related deaths^1^,^26^. The Wnt/β-catenin pathway is mutated in about 90% cases of sporadic and hereditary colorectal cancer^1^,^26^. Abnormal activation of the Wnt pathway promotes nuclear translocation of β-catenin, and increases initiation and maintenance of colon cancers^1^. Nuclear translocation of β-catenin is regulated by several oncogenes, including FoxM1^2^,^3^. FoxM1 promotes the nuclear translocation of β-catenin, thereby increases the expression of β-catenin target genes^3^. Elevated expression of FoxM1 has been detected in most cancers from early tumor development to metastasis^6^,^7^. Elevation in FoxM1 also promotes initiation and maintenance of cancer through regulation of the cancer cell cycle, proliferation, angiogenesis, anti-apoptosis, and invasion^6^,^7^. Targeting FoxM1 is a good strategy for cancer therapeutics, because FoxM1 also regulates Wnt/β-catenin pathway.

In this study, DFS isolated from *A. japonica* suppressed TOPFlash activity (Fig. 1) and suppressed β-catenin nuclear translocation and expression of its target genes in both APC (SW480) or β-catenin (HCT116) mutated colon cancer cells (Fig. 3). Consequently, DFS suppressed colon cancer cell viability and cell cycle progression (Fig. 2) at 10 μM, while normal fibroblast cells (CCD-18Co and MEF cells) were quite resistant to cytotoxicity of DFS. DFS decreased the protein level of FoxM1 without changing the mRNA level in colon cancer cells (Fig. 3E). The FoxM1 destabilizing activity of DFS was also confirmed in cycloheximide-treated colon cancer cells (Fig. 4A,B). DFS decreased the etoposide stabilized FoxM1 level, which is the basis of drug resistance^20^,^21^. Thus, DFS might sensitize cancer cells to chemotherapy^27^. As degradation of FoxM1 depends on proteasomes^22^, siRNA knock-down of proteasomal proteins suppressed FoxM1 degradation (Fig. 4E,F). However, DFS reduced FoxM1 protein levels in proteasomal protein knock-down colon cancer cells (Fig. 4F). We also showed that FoxM1 protein degradation is 20S, 19S, and 11S proteasomal-dependent (Fig. 4F). These results suggest that DFS might have proteasomal independent mechanism for FoxM1 degradation.

DFS-induced FoxM1 protein degradation was blocked by the lysosomal inhibitors chloroquine and bafilomycin A1 (Fig. 5A, S8), indicating that DFS-induced FoxM1 degradation may be dependent on lysosome. This is a novel finding. The lysosomal degradation of FoxM1 might be related with autophagic regulation by DFS. This will need to be investigated in other studies. Autophagy can be tumor-promoting and tumor-suppressing depending on the stage or the type of cancers^28^. Presently, DFS mediated FoxM1 degradation by activating lysosomal-mediated degradation, which reduced the viability of colon cancer cells in culture (Fig. 2) and *in vivo* in a mouse xenograft model (Fig. 6). Although DFS might induce autophagy, and so helping promote tumorigenesis in a context dependent manner, our results suggest that DFS, at least in our colon cancer model, has anti-cancer activity. The lysosomal- dependent regulation of FoxM1 by chemicals might be a new strategy for down-regulating oncogenic transcription factor FoxM1 as a strategy to combat cancer.

Transcriptional regulation of FoxM1 was reported by the direct binding of small molecules, such as FDI-6^29^. FoxM1 harbors an auto-regulation loop, self-binding to its own promoter induces its own expression^30^. This loop can be blocked by inhibiting proteasomal degradation of negative regulator of FoxM1 (NRFM). The transcriptional level of FoxM1 can be down-regulate by the antibiotics siomycin A^31^ and thiostrepton^32^ by inhibition of proteasomal degradation of NRFM^33^. FoxM1 knockdown sensitizes cancer cells to proteasome inhibitor-induced apoptosis but not to autophagy^34^.

In conclusion, DFS from *A. japonica* suppresses colon cancer cell proliferation by reducing β-catenin nuclear translocation. DFS induces lysosomal-dependent FoxM1 protein degradation. This is the first report on the lysosomal degradation of FoxM1 by a small molecule. DFS may be useful in treating cancers that feature the elevated expression of FoxM1.

## Methods

### Reagents

*A. japonica* was purchased from Yeongcheon, Gyungsangbuk-do, Korea in March, 2012. Cyclin D1, lamin A/C, c-Myc, and survivin antibodies were purchased from Cell Signaling Technology (Danvers, MA, USA), β-catenin was purchased from BD Bioscience (San Jose, CA, USA), FoxM1 was purchased from Bethyl Laboratories (Montgomery, TX, USA), β-actin antibody and 3-(4,5-dimethylthiazol-2-yl)-2,5-diphenyl-tetrazolium bromide (MTT) were purchased from Sigma-Aldrich (St. Louis, MO, USA). Horseradish peroxidase-conjugated anti-rabbit immunoglobulin G (IgG) and anti-mouse IgG antibodies were purchased from Assay Designs (Ann Arbor, MI, USA). Lipofectamine LTX PLUS was purchased from Life Technology (Carlsbad, CA, USA). The luciferase assay system was purchased from Promega (Madison, WI, USA). Enhanced chemiluminescence agent was purchased from GE Healthcare Life Science (Uppsala, Sweden).

### Extraction and isolation of DFS

*A. japonica* stems (5.8 kg) were extracted with methanol (MeOH) under reflux, and the concentrated extracts (294 g) were partitioned with chloroform (CHCl_3_) to yield a CHCl_3_-soluble fraction. The CHCl_3_ fraction (28 g) was subjected to silica gel column chromatography using hexane/ethyl acetate as an eluent to obtain three fractions. Further chromatography of fraction 2 (1.7 g) using reverse phase chromatography (gradient elution from 30% MeOH to 80% MeOH) yielded DFS (180 mg), and its purity was confirmed by high performance liquid chromatography and ^1^H-nuclear magnetic resonance spectrum.

### Cell culture

The human colorectal adenocarcinoma SW480 cell line and normal colon fibroblast cell line CCD-18Co cells were purchased from the Korean Cell Line Bank (Seoul, Korea). Mouse embryonic fibroblast (MEF) was prepared from embryos (day 12.5) of C57BL/6 mouse as described previously^35^. The HCT116 human colorectal carcinoma cell line, 293 T cell line, and Wnt3a-secreting L cell line (L-Wnt3a) were purchased from the American Type Culture Collection (Manassas, VA, USA). The HEK293 reporter cell line (stably transfected with TOPFlash, a synthetic β-catenin/TCF-dependent luciferase reporter, and human Frizzled-1 expression plasmids) and a control cell line (stably transfected with FOPFlash, a negative control reporter with mutated β-catenin/TCF binding elements)^36^ were kindly provided by Prof. Sangtaek Oh, Kookmin University (Seoul, Korea). SW480 and CCD-18Co cells were cultured in RMPI. The other cells were cultured in Dulbecco’s Modified Eagle Medium (DMEM) containing 10% fetal bovine serum (FBS), penicillin (100 U/ml), and streptomycin (10 μg/ml) at 37 °C.

### Preparation of Wnt3a conditioned medium (CM)

Wnt3a CM and control CM were prepared as previously described^37^. L-Wnt3a cells or L-control cells (1.0 × 10^6^ cells in a 100 mm diameter dish) were cultured in DMEM containing 10% FBS. After 4 days, the medium was collected to obtain the first batch of CM. Fresh medium was added, and the cells were cultured for another 3 days at 37 °C, at which point a second batch of CM was collected. The collected medium was passed through a sterile 0.20 μm membrane filter. Wnt3a CM and control CM were prepared by combining equal volumes of the first and second batches of media.

### Cloning of FoxM1 and adenovirus preparation

FoxM1B and FoxM1C were cloned from Huh7 hepatoma cells and cloned into the pcDNA3 vector (Promega)^38^. FoxM1B cDNA was inserted into replication-defective E1- and E3-adenoviral vectors. FoxM1B adenovirus was then amplified in HEK293 human kidney epithelial cells, and the viral particles were purified by passage through a 0.45 μm pore size filter. Similarly, a bacterial β-galactosidase adenovirus was prepared as a control (LacZ).

### Reporter gene assay

TOPFlash- or FOPFlash-transfected SW480 and HCT116 cells and TOPFlash reporter- or control FOPFlash reporter-expressing HEK293 cells were treated with the indicated concentrations of DFS for 16 h. Luciferase activity was determined with a luminometer (Promega) using a luciferase assay system (Promega). The results were normalized by the activity of FOPFlash, a negative control reporter with mutated β-catenin/Tcf binding elements.

### Western blotting analysis

Cells were washed with ice-cold phosphate buffered saline and lysed with lysis buffer (1% NP-40, 0.1% sodium dodecyl sulfate, 150 mM sodium chloride, and 50 mM Tris, pH 7.4) to obtain whole-cell lysates. The cytosolic and nuclear fractions were prepared with a nuclear and cytosol extraction kit (Thermo Scientific, Rockford, IL, USA) according to the manufacturer’s instructions. Proteins in the lysates were resolved by sodium dodecyl sulfate-polyacrylamide gel electrophoresis and transferred to polyvinylidene difluoride membranes. The bands were detected by enhanced chemiluminescence after incubation with the indicated primary and secondary antibodies. The membranes were reprobed after removal of antibodies with stripping buffer (2% sodium dodecyl sulfate, 62.5 mM Tris-HCl, pH 6.8, 0.8% β-mercaptoethanol) for 30 min at 50 °C.

### Reverse-transcription polymerase chain reaction (PCR)

Cells were treated with the indicated concentrations of DFS for 16 h. Total RNA was isolated from the cell pellets using TRIzol Reagent (Life Technology), and reverse transcribed to single-stranded cDNA using the SuperScript II first-strand cDNA synthesis system according to the manufacturer’s instructions. cDNA was amplified using a PCR thermal cycler. The PCR primer sequences were as follows: FoxM1, sense 5′-CCTTGTGTTCCCCAAGAGTAT-3′ and anti-sense 5′-CTGCAGGTCTTCCCTTCTATC-3′. Beta-actin was amplified as an internal control. The amplified DNA was separated on 2% agarose gels containing 0.01% ethidium bromide.

### Cell viability and cell cycle analysis

Cell viability was determined by the MTT assay as described previously^37^. The cells were treated with the indicated concentrations of DFS. The cells were incubated with 50 μg/ml MTT, formazan crystals were dissolved with dimethylsulfoxide, and the absorbance was measured at 540 nm using a Versa Max microplate reader (Molecular Devices, Sunnyvale, CA, USA) at 24, 48 and 72 h.

To analyze the cell cycle, the cells were treated with 20 μM DFS for 24 and 48 h, fixed with 70% ethanol and stained with propidium iodide. The stained cells were analyzed by fluorescence-activated cell sorting (FACS, BD Biosciences, San Jose, CA, USA).

### Tumor xenograft model

SW480 cells (9 × 10^6^ cells/mouse) were suspended in Matrigel^®^ (Corning, Tewksbury, MA, USA) and inoculated subcutaneously in the right flank of 5-week old female BALB/c nu/nu mice (Nara Biotech, Seoul, Republic of Korea). When tumor volume was ~40 mm^3^, mice were randomly distributed and treated with vehicle (10% dimethylacetamide, 5% Tween 80 and 85% [20% hydroxypropyl β -cyclodextrin]), 30 mg/kg of DFS or 2 mg/kg of doxorubicin by intraperitoneal injection (n = 5). Mice were treated with vehicle or DFS 5 days a week and with doxorubicin every other day for 3 weeks. Tumor volumes were estimated as length (mm) × width (mm) × height (mm)/2. On day 21, mice were sacrificed and tumor weights were measured. All animal experiment was conducted by protocols approved (Approval #: KRIBB-AEC-16001) by Institutional Animal Care and Use Committee at Korea Research Institute of Bioscience and Biotechnology (KRIBB, Daejeon, Korea), and was performed in accordance with the Guide for Care and Use of Laboratory Animals of KRIBB.

### Statistical analysis

IC_50_ values were calculated using the GraphPad Prism computer program (GraphPad Software, Inc., La Jolla, CA, USA). Data are expressed as mean ±standard deviation. All experiments were performed at least four times. Statistical analysis was performed using the Student’s *t*-test. A *p* value < 0.01 was considered statistically significant.

## Additional Information

**How to cite this article:** Dong, G.-Z. *et al*. A lignan induces lysosomal dependent degradation of FoxM1 protein to suppress β-catenin nuclear translocation. *Sci. Rep.* **7**, 45951; doi: 10.1038/srep45951 (2017).

**Publisher's note:** Springer Nature remains neutral with regard to jurisdictional claims in published maps and institutional affiliations.

## Supplementary Material

Supplementary Information

## References

[b1] Miyaki M. . Characteristics of somatic mutation of the adenomatous polyposis coli gene in colorectal tumors. Cancer Res. 54, 3011–3020 (1994). 8187091

[b2] Morgan R. G., Ridsdale J., Tonks A. & Darley R. L. Factors affecting the nuclear localization of beta-catenin in normal and malignant tissue. J. Cell. Biochem. 115, 1351–1361 (2014). 24610469 10.1002/jcb.24803

[b3] Zhang N. . FoxM1 promotes beta-catenin nuclear localization and controls Wnt target-gene expression and glioma tumorigenesis. Cancer Cell 20, 427–442 (2011). 22014570 10.1016/j.ccr.2011.08.016 PMC3199318

[b4] Behrens J. . Functional interaction of [beta]-catenin with the transcription factor LEF-1. Nature 382, 638–642 (1996). 8757136 10.1038/382638a0

[b5] Sparks A. B., Morin P. J., Vogelstein B. & Kinzler K. W. Mutational analysis of the APC/β-catenin/Tcf pathway in colorectal cancer. Cancer Res. 58, 1130–1134 (1998). 9515795

[b6] Halasi M. & Gartel A. L. FOX(M1) news–it is cancer. Mol. Cancer Ther. 12, 245–254 (2013). 23443798 10.1158/1535-7163.MCT-12-0712 PMC3596487

[b7] Pilarsky C., Wenzig M., Specht T., Saeger H. D. & Grutzmann R. Identification and validation of commonly overexpressed genes in solid tumors by comparison of microarray data. Neoplasia 6, 744–750 (2004). 15720800 10.1593/neo.04277 PMC1531678

[b8] Yoshida Y., Wang I. C., Yoder H. M., Davidson N. O. & Costa R. H. The forkhead box M1 transcription factor contributes to the development and growth of mouse colorectal cancer. Gastroenterology 132, 1420–1431 (2007). 17408638 10.1053/j.gastro.2007.01.036

[b9] Kuroyanagi M. . New diarylheptanoids from Alnus japonica and their antioxidative activity. Chem. Pharm. Bull. 53, 1519–1523 (2005). 16327181 10.1248/cpb.53.1519

[b10] Tung N. H. . Antioxidative and hepatoprotective diarylheptanoids from the bark of Alnus japonica. Planta Med. 76, 626–629 (2010). 19918716 10.1055/s-0029-1240595

[b11] Tung N. H. . Anti-influenza diarylheptanoids from the bark of Alnus japonica. Bioorg. Med. Chem. Lett. 20, 1000–1003 (2010). 20045319 10.1016/j.bmcl.2009.12.057

[b12] Tung N. H. . An anti-influenza component of the bark of Alnus japonica. Arch. Pharm. Res. 33, 363–367 (2010). 20361299 10.1007/s12272-010-0303-5

[b13] Uto T. . Antiproliferative and pro-apoptotic activity of diarylheptanoids isolated from the bark of Alnus japonica in human leukemia cell lines. Am. J. Chin. Med. **43**, 757–767 (2015). 26119959 10.1142/S0192415X15500470

[b14] Dong G.-z. . Diarylheptanoids from lesser galangal suppress human colon cancer cell growth through modulating Wnt/β-catenin pathway. J. Funct. Foods 18, Part A, 47–57 (2015).

[b15] Nomura M., Tokoroyama T. & Kubota T. Further phenolic components from Alnus japonica Steud. J. Chem. Soc. Chem. Commun. 316–317 (1975).

[b16] Moon S. S., Rahman A. A., Kim J. Y. & Kee S. H. Hanultarin, a cytotoxic lignan as an inhibitor of actin cytoskeleton polymerization from the seeds of Trichosanthes kirilowii. Bioorg. Med. Chem. 16, 7264–7269 (2008). 18603435 10.1016/j.bmc.2008.06.032

[b17] Wu P. L., Chuang T. H., He C. X. & Wu T. S. Cytotoxicity of phenylpropanoid esters from the stems of Hibiscus taiwanensis. Bioorg. Med. Chem. 12, 2193–2197 (2004). 15080919 10.1016/j.bmc.2004.02.020

[b18] Yan J. . Identification of two novel inhibitors of mTOR signaling pathway based on high content screening. Cancer Chemother. Pharmacol. 72, 799–808 (2013). 23934262 10.1007/s00280-013-2255-1

[b19] Bienz M. & Clevers H. Linking colorectal cancer to Wnt signaling. Cell 103, 311–320 (2000). 11057903 10.1016/s0092-8674(00)00122-7

[b20] Tan Y., Raychaudhuri P. & Costa R. H. Chk2 mediates stabilization of the FoxM1 transcription factor to stimulate expression of DNA repair genes. Mol. Cell. Biol. 27, 1007–1016 (2007). 17101782 10.1128/MCB.01068-06 PMC1800696

[b21] Tan Y. . Two-fold elevation of expression of FoxM1 transcription factor in mouse embryonic fibroblasts enhances cell cycle checkpoint activity by stimulating p21 and Chk1 transcription. Cell Prolif. 43, 494–504 (2010). 20887555 10.1111/j.1365-2184.2010.00699.x PMC6496485

[b22] Myatt S. S. . SUMOylation inhibits FOXM1 activity and delays mitotic transition. Oncogene 33, 4316–4329 (2014). 24362530 10.1038/onc.2013.546 PMC4096495

[b23] Bhat U. G., Halasi M. & Gartel A. L. FoxM1 is a general target for proteasome inhibitors. PloS One 4, e6593 (2009). 19672316 10.1371/journal.pone.0006593 PMC2721658

[b24] Jia L., Yu G., Zhang Y. & Wang M. M. Lysosome-dependent degradation of Notch3. Int. J. Biochem. Cell Biol. 41, 2594–2598 (2009). 19735738 10.1016/j.biocel.2009.08.019 PMC2811749

[b25] Palacios F., Tushir J. S., Fujita Y. & D’Souza-Schorey C. Lysosomal targeting of e-cadherin: a unique mechanism for the down-regulation of cell-cell adhesion during epithelial to mesenchymal transitions. Mol. Cell. Biol. 25, 389–402 (2005). 15601859 10.1128/MCB.25.1.389-402.2005 PMC538771

[b26] Jemal A. . Global cancer statistics. CA Cancer J. Clin. 61, 69–90 (2011). 21296855 10.3322/caac.20107

[b27] Halasi M. & Gartel A. L. Suppression of FOXM1 sensitizes human cancer cells to cell death induced by DNA-damage. PloS One 7, e31761 (2012). 22393369 10.1371/journal.pone.0031761 PMC3290538

[b28] Janku F., McConkey D. J., Hong D. S. & Kurzrock R. Autophagy as a target for anticancer therapy. Nat. Rev. Clin. Oncol. 8, 528–539 (2011). 21587219 10.1038/nrclinonc.2011.71

[b29] Gormally M. V. . Suppression of the FOXM1 transcriptional programme via novel small molecule inhibition. Nat. Commun. 5, 5165 (2014). 25387393 10.1038/ncomms6165 PMC4258842

[b30] Halasi M. & Gartel A. L. A novel mode of FoxM1 regulation: positive auto-regulatory loop. Cell Cycle 8, 1966–1967 (2009). 19411834 10.4161/cc.8.12.8708

[b31] Radhakrishnan S. K. . Identification of a chemical inhibitor of the oncogenic transcription factor forkhead box M1. Cancer Res. 66, 9731–9735 (2006). 17018632 10.1158/0008-5472.CAN-06-1576

[b32] Kwok J. M. . Thiostrepton selectively targets breast cancer cells through inhibition of forkhead box M1 expression. Mol. Cancer Ther. 7, 2022–2032 (2008). 18645012 10.1158/1535-7163.MCT-08-0188

[b33] Gartel A. L. Thiostrepton, proteasome inhibitors and FOXM1. Cell Cycle 10, 4341–4342 (2011). 22134246 10.4161/cc.10.24.18544 PMC3272263

[b34] Pandit B. & Gartel A. L. FoxM1 knockdown sensitizes human cancer cells to proteasome inhibitor-induced apoptosis but not to autophagy. Cell Cycle 10, 3269–3273 (2011). 21941087 10.4161/cc.10.19.17735 PMC3233624

[b35] Kim K. I. . Ube1L and protein ISGylation are not essential for alpha/beta interferon signaling. Mol. Cell. Biol. 26(2), p.472–9 (2006). 16382139 10.1128/MCB.26.2.472-479.2006 PMC1346917

[b36] Park S. . Hexachlorophene inhibits Wnt/beta-catenin pathway by promoting Siah-mediated beta-catenin degradation. Mol. Pharmacol. 70, 960–966 (2006). 16735606 10.1124/mol.106.024729

[b37] Dong G. z., Shim A. R., Hyeon J. S., Lee H. J. & Ryu J. H. Inhibition of Wnt/beta-catenin pathway by dehydrocostus lactone and costunolide in colon cancer cells. Phytother. Res. 29, 680–686 (2015). 25625870 10.1002/ptr.5299

[b38] Park T. J. . TIS21 negatively regulates hepatocarcinogenesis by disruption of cyclin B1-Forkhead box M1 regulation loop. Hepatology 47, 1533–1543 (2008). 18393292 10.1002/hep.22212

