# Supplementary material for: A lignan induces lysosomal dependent degradation of FoxM1 protein to suppress β-catenin nuclear translocation
Source: Sci Rep. 2017 Apr 5;7:45951. doi: 10.1038/srep45951 (PMC5380986; doi:10.1038/srep45951)
Supplement: Supplementary Information [file srep45951-s1.pdf]

Supplementary Information

A lignan induces lysosomal dependent degradation of FoxM1 protein to suppress β-catenin nuclear translocation

Guang-Zhi Dong[1], Ji Hye Jeong[1], Yu-ih Lee[1], Yeong Eun Han[1], Jung Sook Shin[1], Yoon-Jung Kim[1], Raok Jeon[1], Young Hwa Kim[2], Tae Jun Park[2], Keun Il Kim[3], Jae-Ha Ryu[1*]

[1] Research Center for Cell Fate Control and College of Pharmacy, Sookmyung Women's University, Seoul 140-742, Korea;

[2] Department of Biochemistry and Molecular Biology, School of Medicine, Ajou University, Suwon, 16499, Korea;

[3] Department of Biological Science, Sookmyung Women's University, Seoul 140-742, Korea

[*] Corresponding Authors: ryuha@sookmyung.ac.kr, College of Pharmacy, Sookmyung Women's University, 100 Chungparo 47 Gil, Yongsan-Gu ,Seoul 140-742, Korea, phone: +82-2-710-9568, fax: +82-2-2077-7869

Supplementary Information

Figure S1. High Resolution QTOF Mass spectrum of DFS ($C_{40}H_{42}O_{12}$)

Figure S2. ^{1}H-NMR spectrum of DFS (400 MHz in DMSO-d_6)

Figure S3. ^{13}C-NMR spectrum of DFS (100 MHz in DMSO-d_6)

Figure S4. ^{1}H-^{1}H COSY spectrum of DFS (400 MHz in DMSO-d_6)

Figure S5. HSQC spectrum of DFS (400 MHz in DMSO-d_6)

Figure S6. HMBC spectrum of DFS (400 MHz in DMSO-d_6)

Figure S7. Fractionation control of nuclear and cytosolic fraction in figure 3.

Figure S8. DFS induces lysosomal dependent degradation of FoxM1.

Figure S9. Xenograft study of DFS

Figure S10. Original image of western blog and RT-PCR.

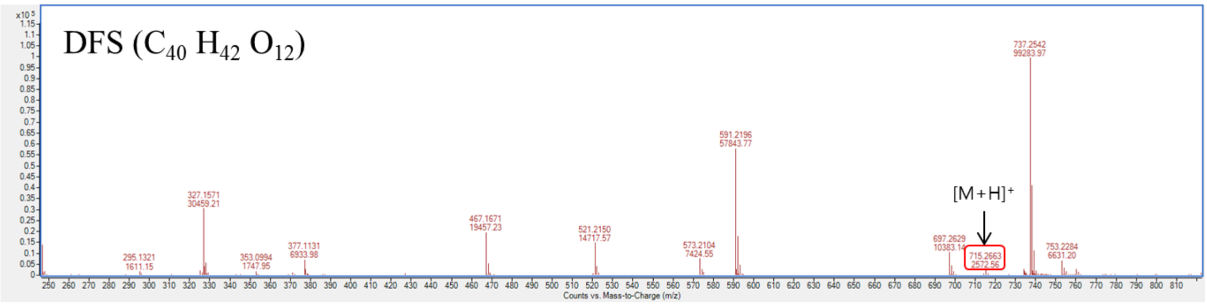

- MW 715.2743 (Theoretical [M+H]$^{+}$)
- MW 715.2663 (Observed [M+H]$^{+}$)

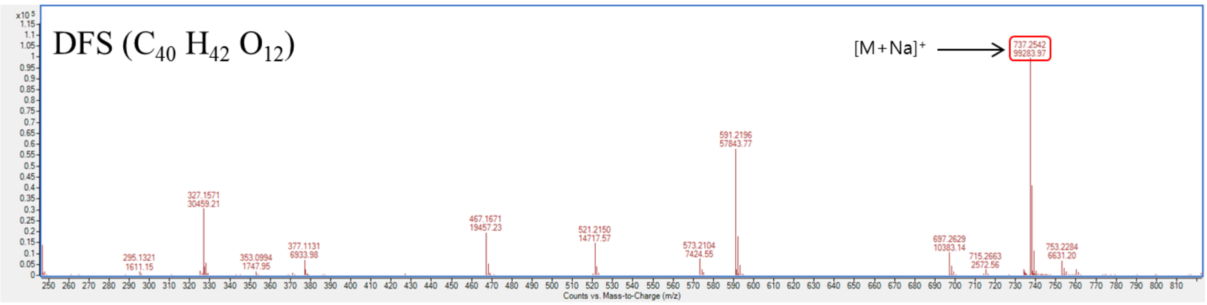

- MW 737.2568 (Theoretical [M+ Na]$^{+}$)
- MW 737.2542 (Observed [M+ Na]$^{+}$)

Figure S1. High Resolution QTOF Mass spectrum of DFS (C_{40} H_{42} O_{12})

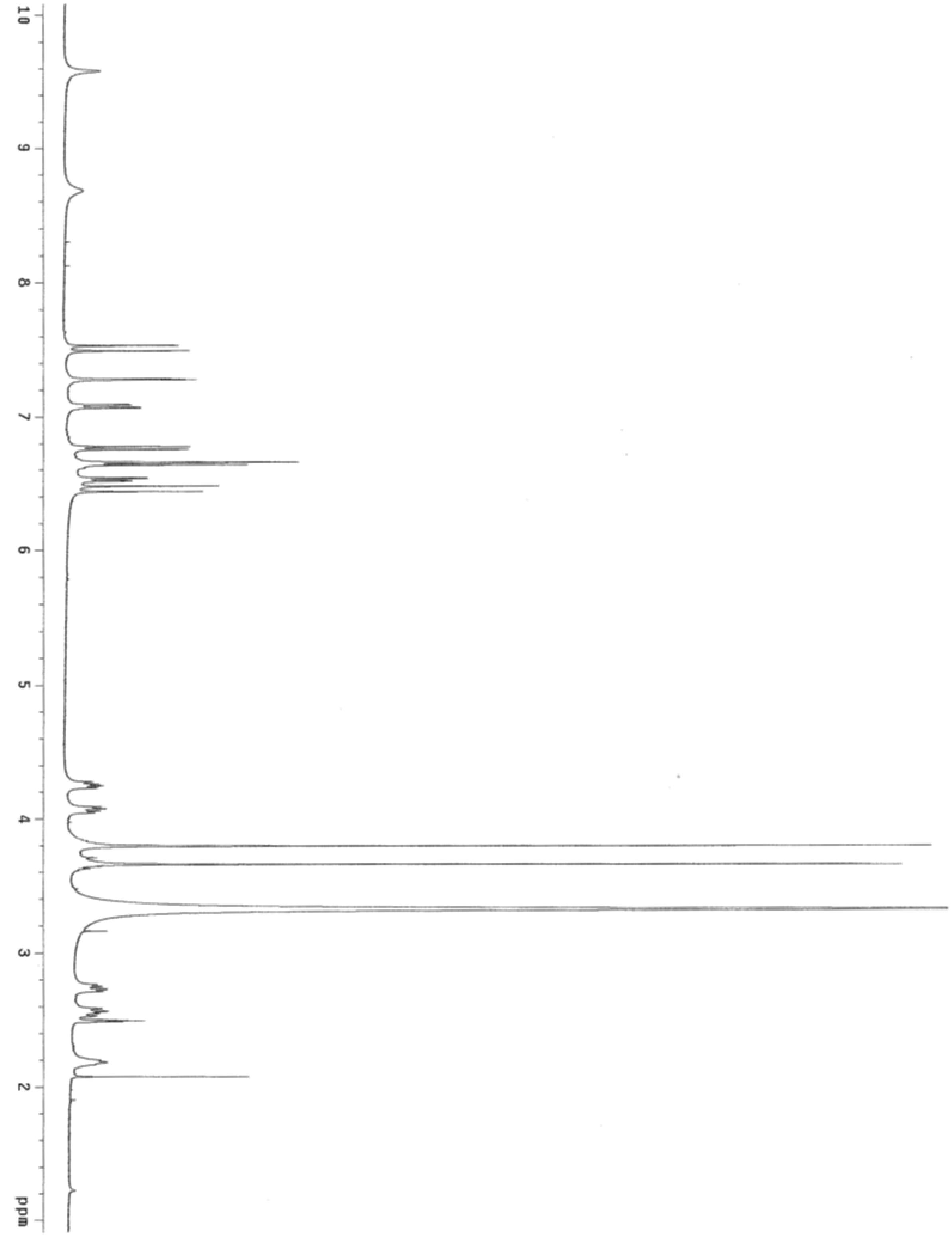

Figure S2. ^{1}H-NMR spectrum of DFS (400 MHz in DMSO-d_6)

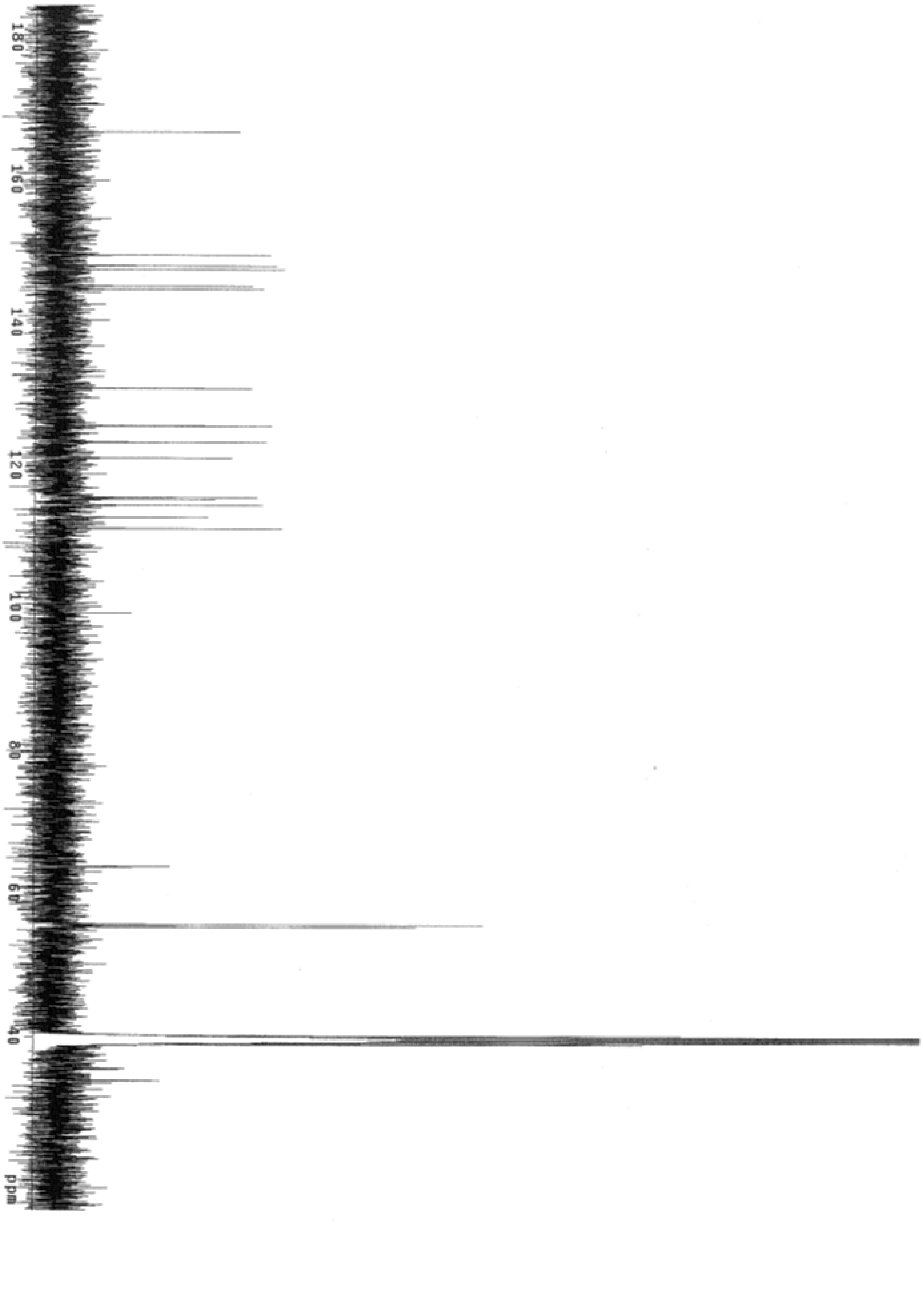

Figure S3. ^{13}C-NMR spectrum of DFS (100 MHz in DMSO-d_6)

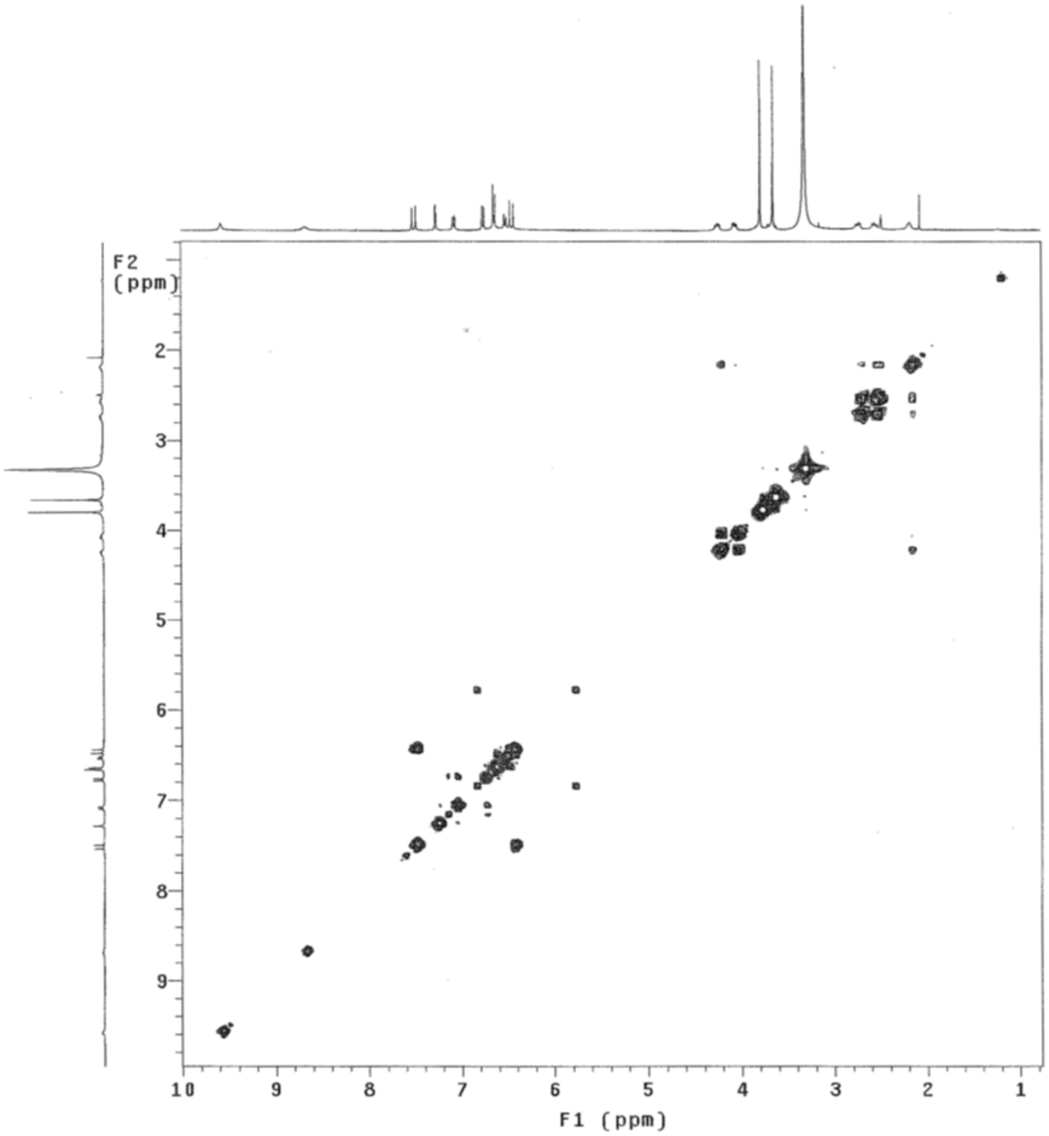

Figure S4. ^{1}H-^{1}H COSY spectrum of DFS (400 MHz in DMSO-d_6)

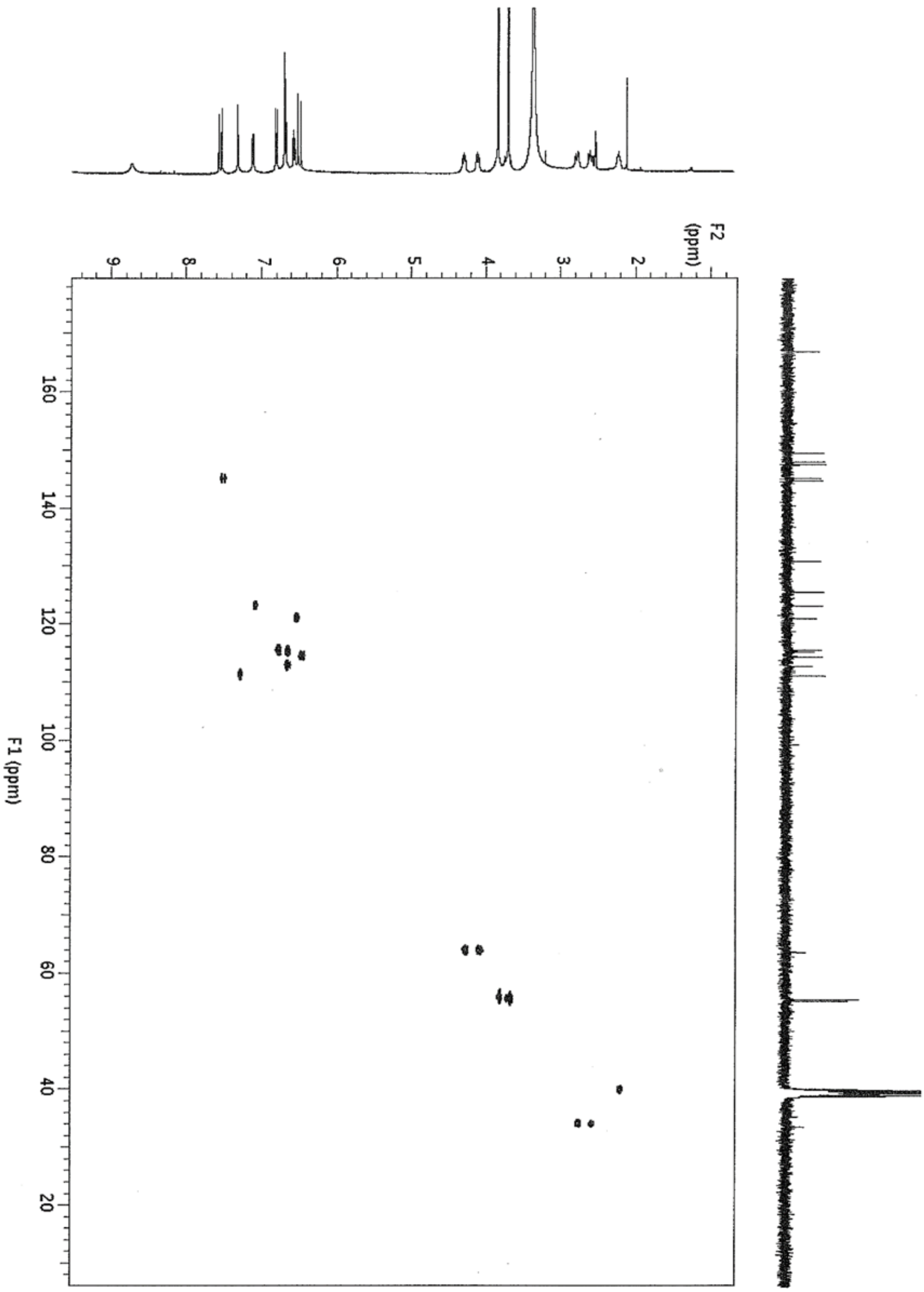

Figure S5. HSQC spectrum of DFS (400 MHz in DMSO-d_6)

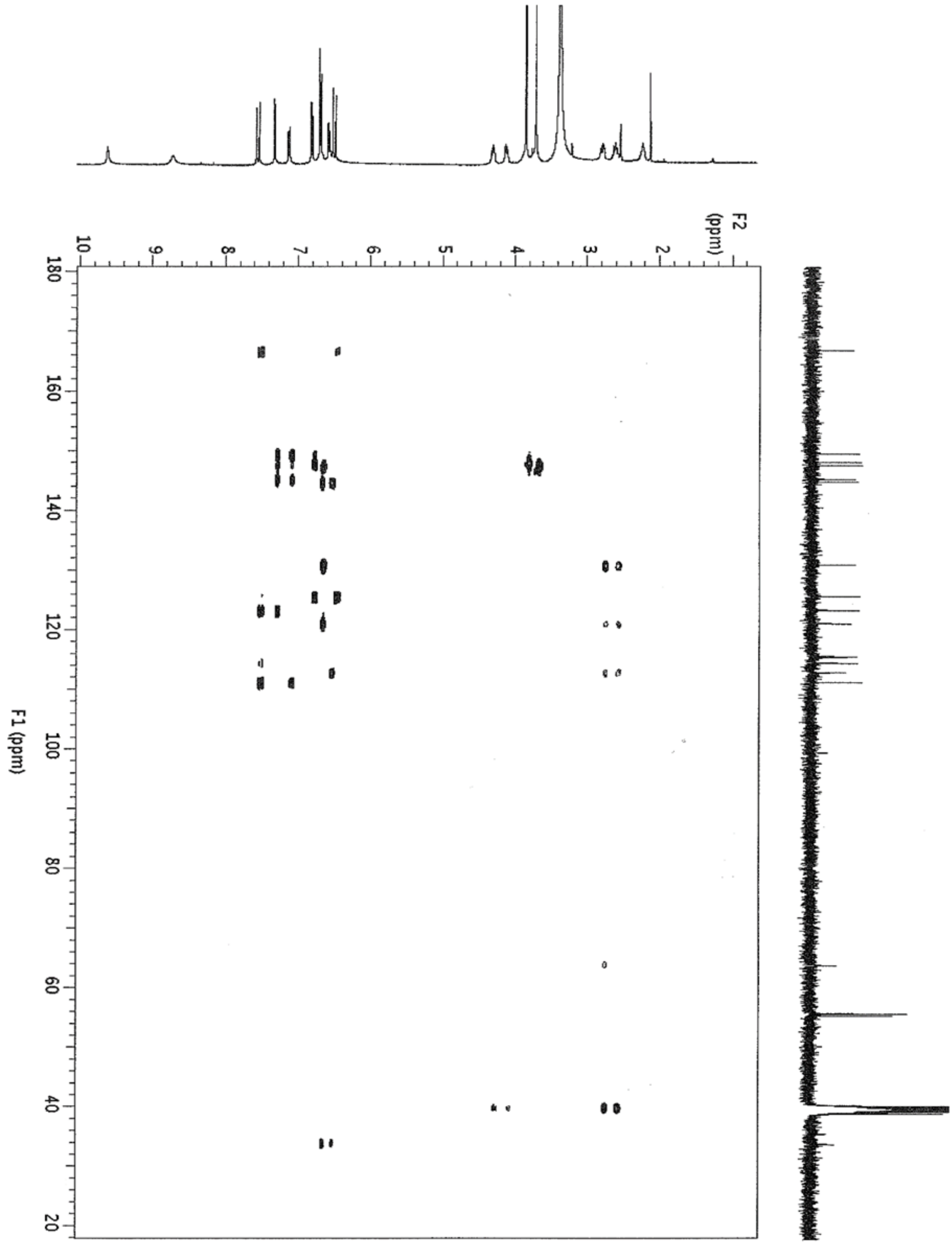

Figure S6. HMBC spectrum of DFS (400 MHz in DMSO-d$_6$)

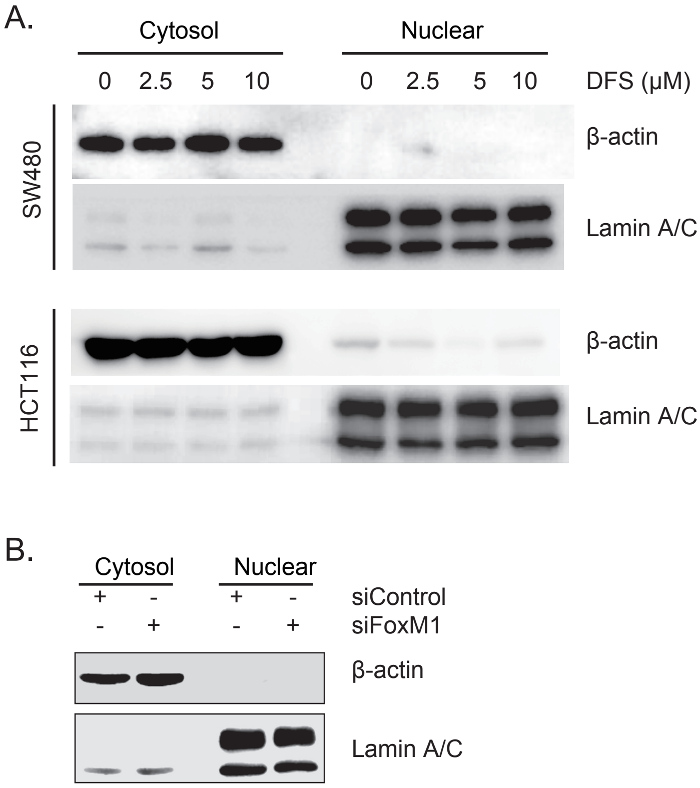

Figure S7. Fractionation control of nuclear and cytosolic fraction in figure 3. SW480 and HCT116 colon cancer cells were treated with the indicated concentrations of DFS for 16 h and the cytosolic and nuclear marker protein levels were determined by Western blot (A). SW480 cells were transfected with siRNA against FoxM1 and the cytosolic and nuclear marker protein levels were determined by Western blot (B).

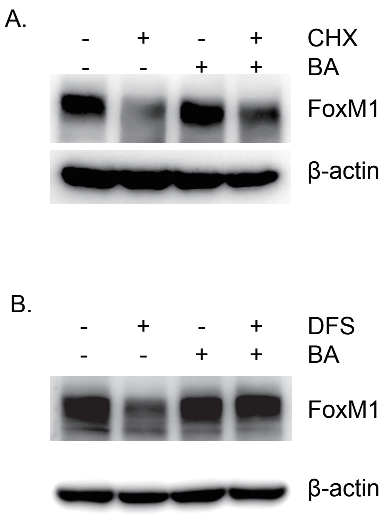

Figure S8. DFS induces lysosomal dependent degradation of FoxM1. Protein levels of FoxM1 were determined by Western blot in SW480 cells after treatment with bafilomycin A1 (BA) and cycloheximide (CHX) (A), or DFS (B).

A.

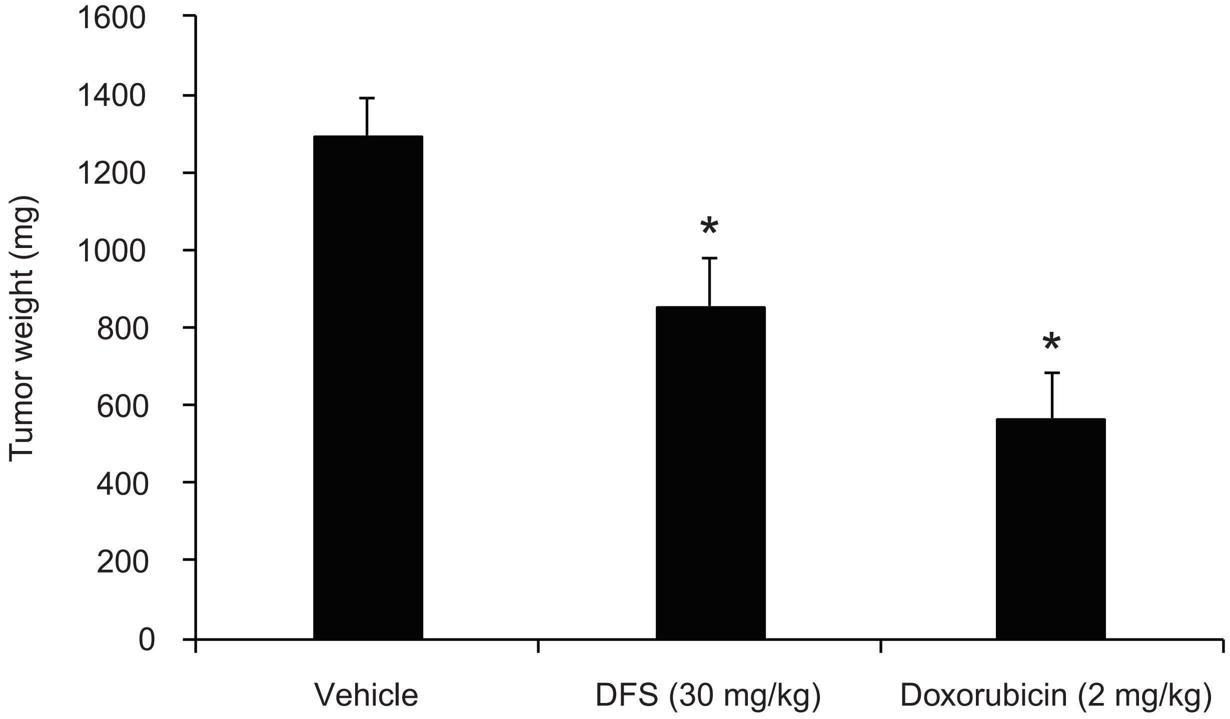

B.

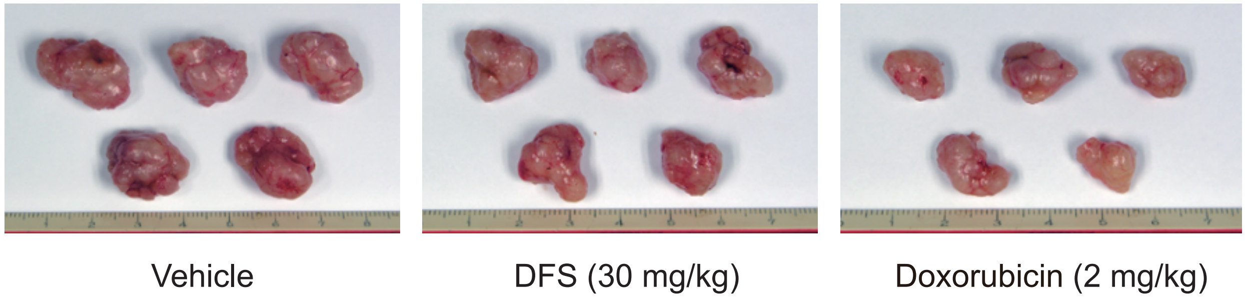

Figure S9. Xenograft study of DFS. SW480 cells (9×10^6 cells/mouse) were inoculated subcutaneously into the right flank of nude mice. Treatment was initiated when tumor volumes reached approximately ~40 mm^3. DFS (30 mg/kg body weight) was administered intraperitonally 5 days a week for 21 days. On day 21, mice were sacrificed and tumor weight was measured (A) and the original image of tumor (B). The asterisk (*) indicated significancy compared to vehicle group ($p < 0.01$).

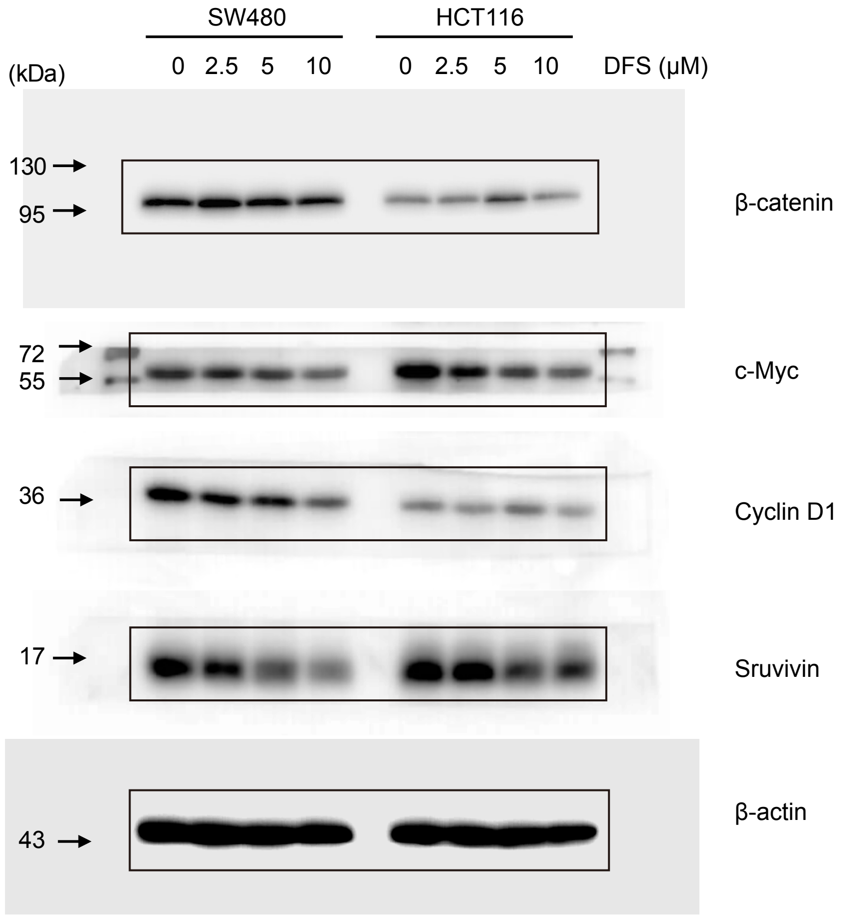

Figure S10a. original image of figure 3A.

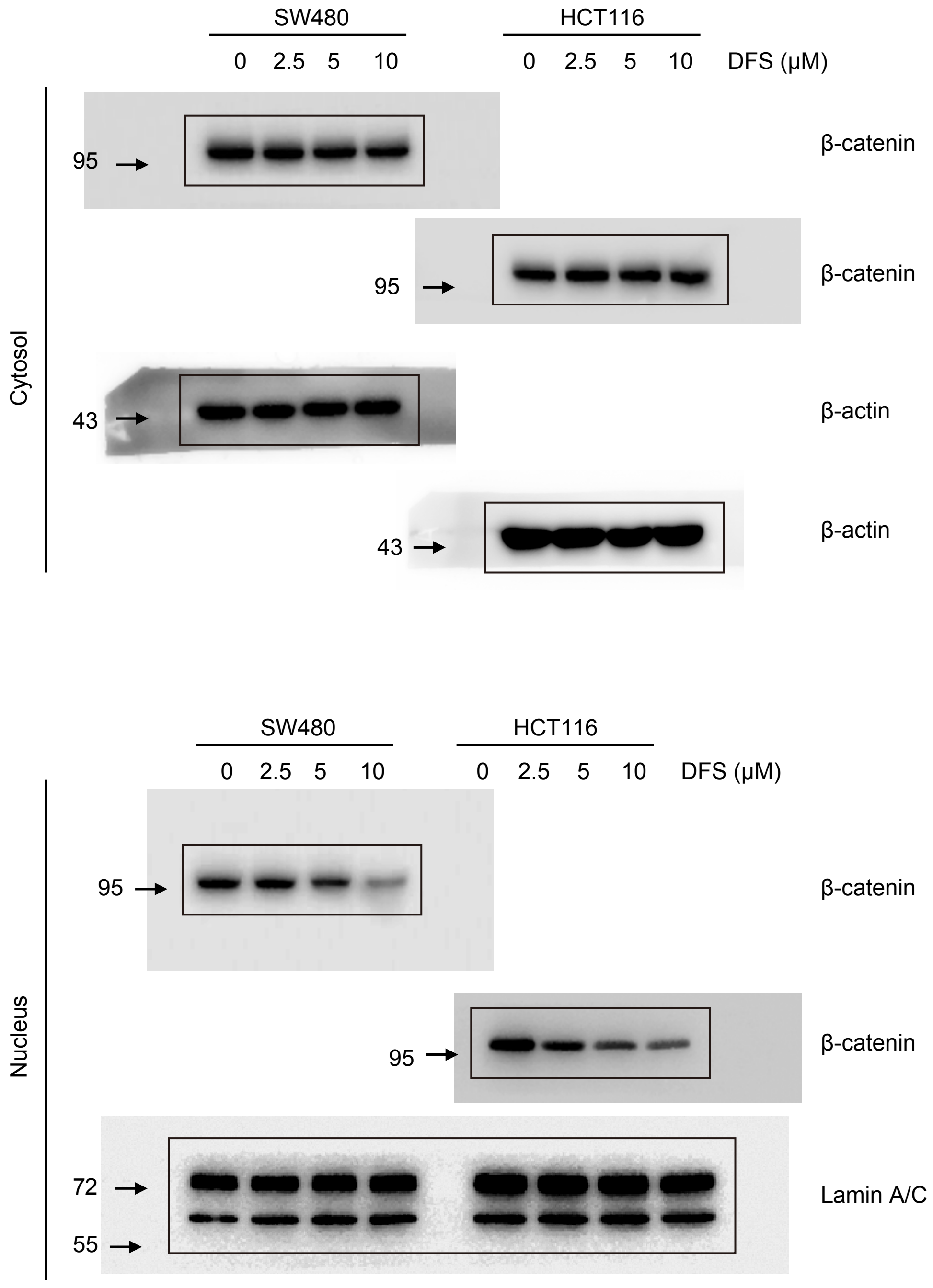

Figure S10b. original image of figure 3B.

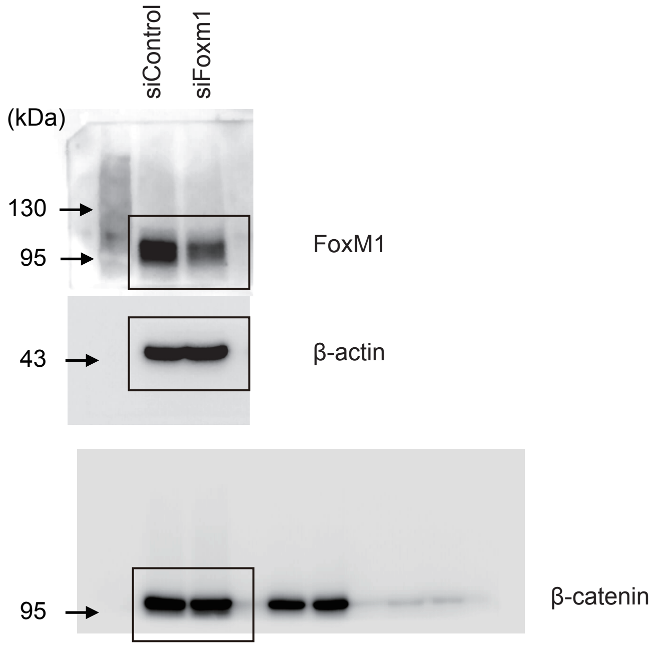

Figure S10c. original image of figure 3C.

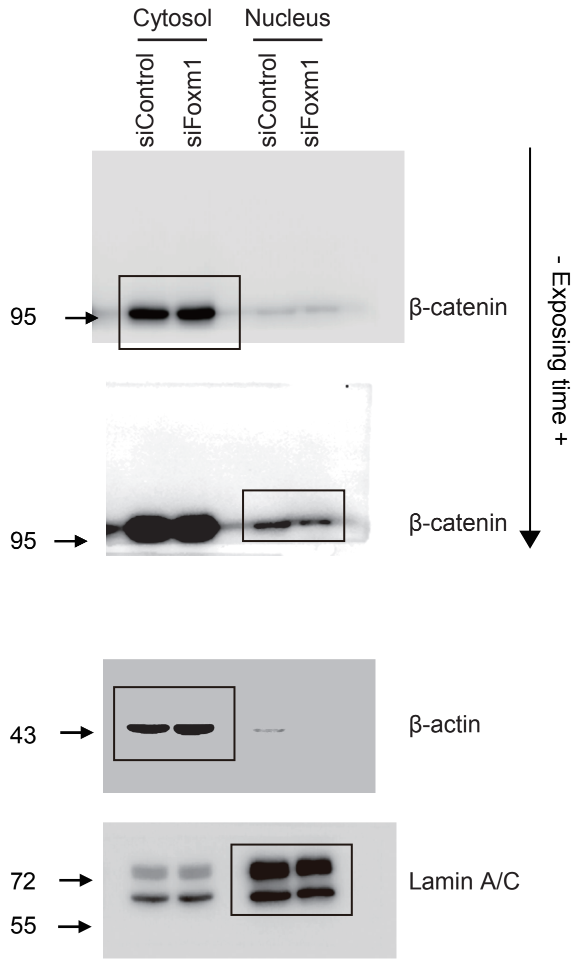

Figure S10d. original image of figure 3D.

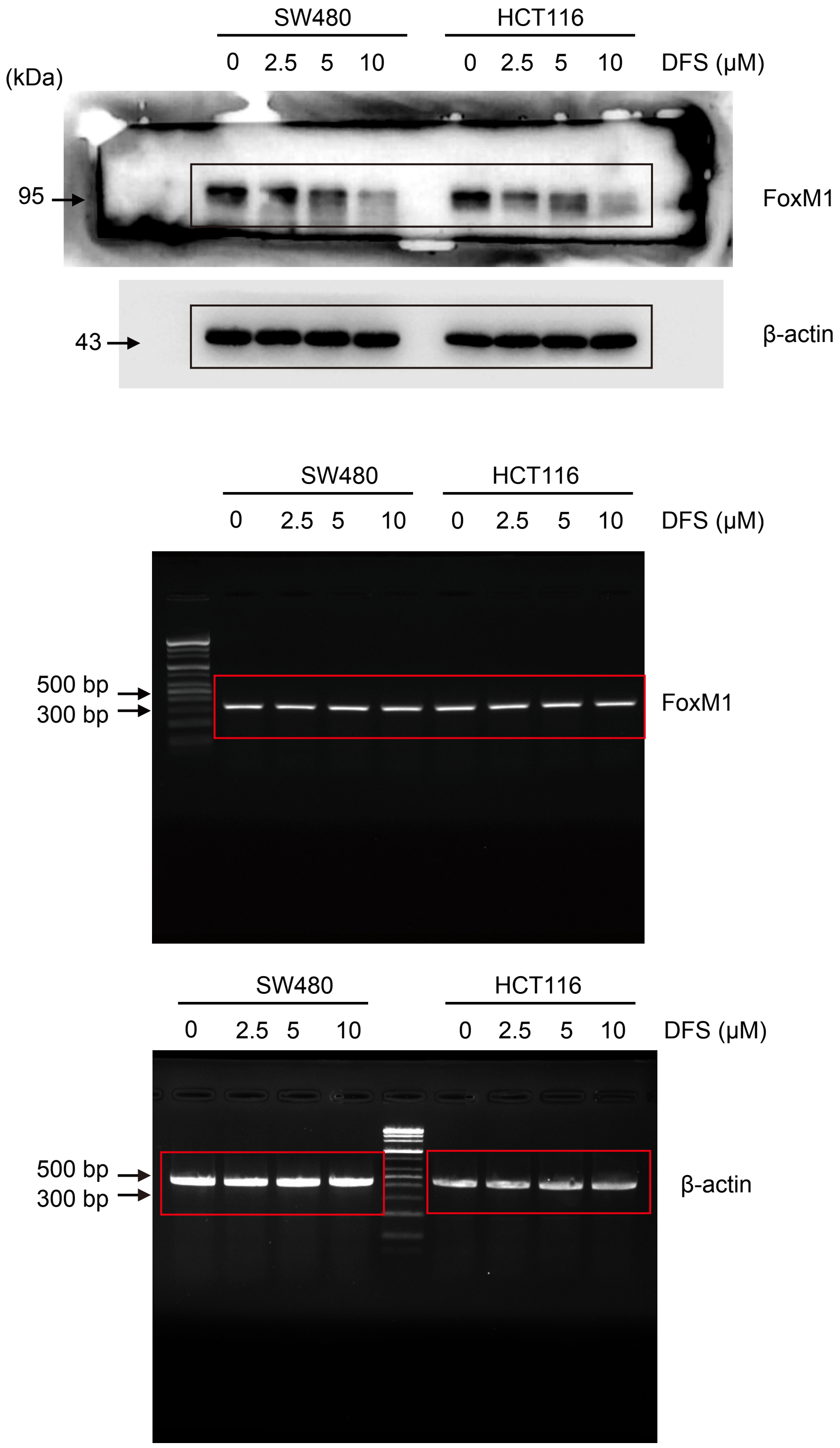

Figure S10e. original image of figure 3E.

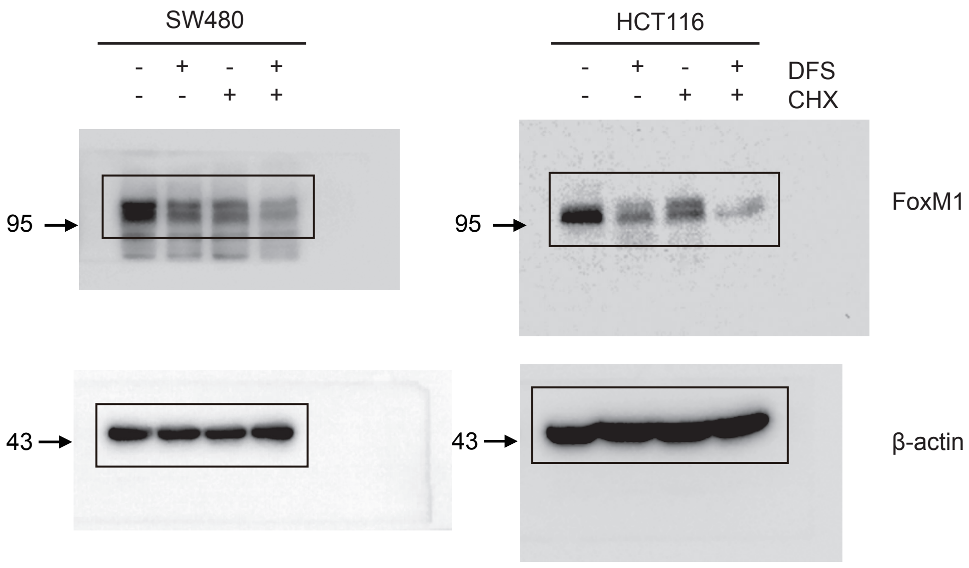

Figure S10f. original image of figure 4A.

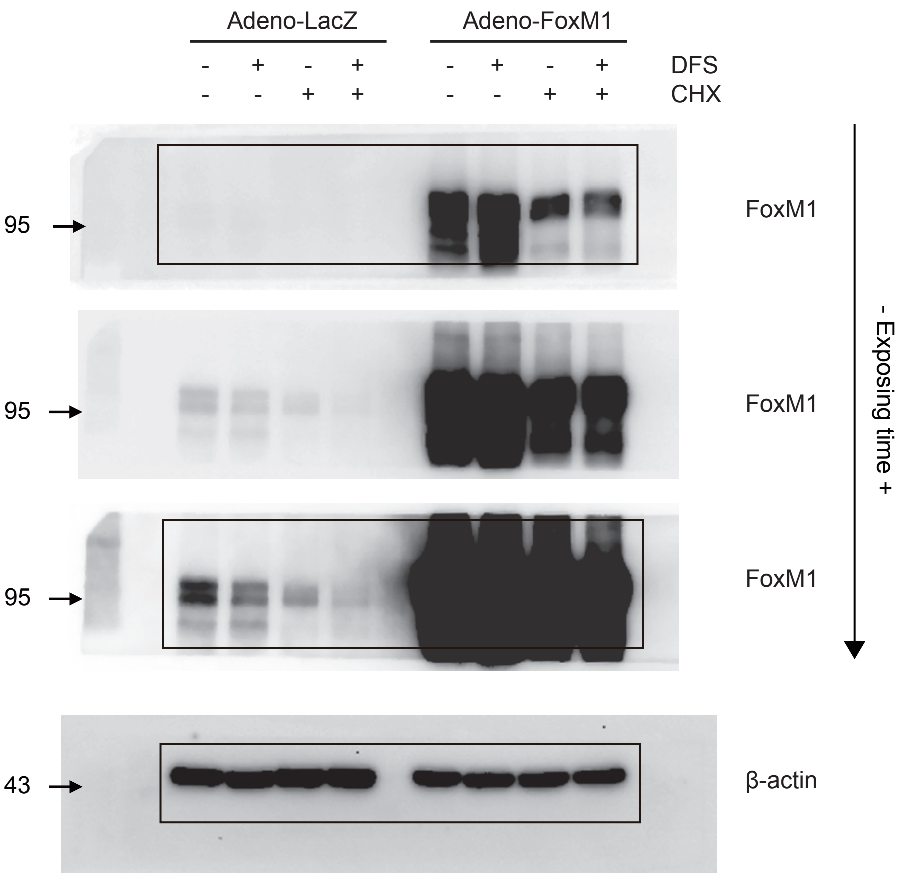

Figure S10g. original image of figure 4B.

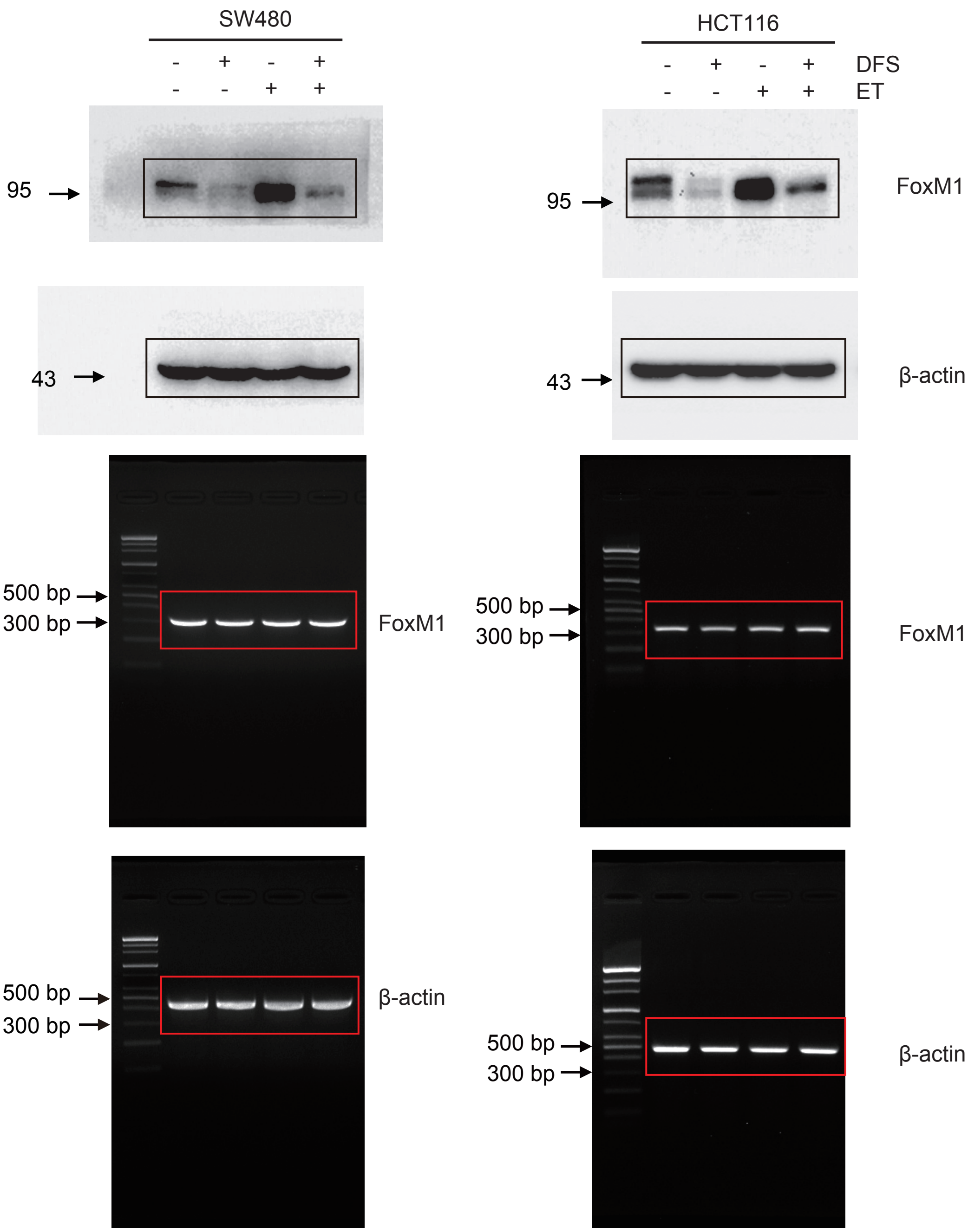

Figure S10h. original image of figure 4C.

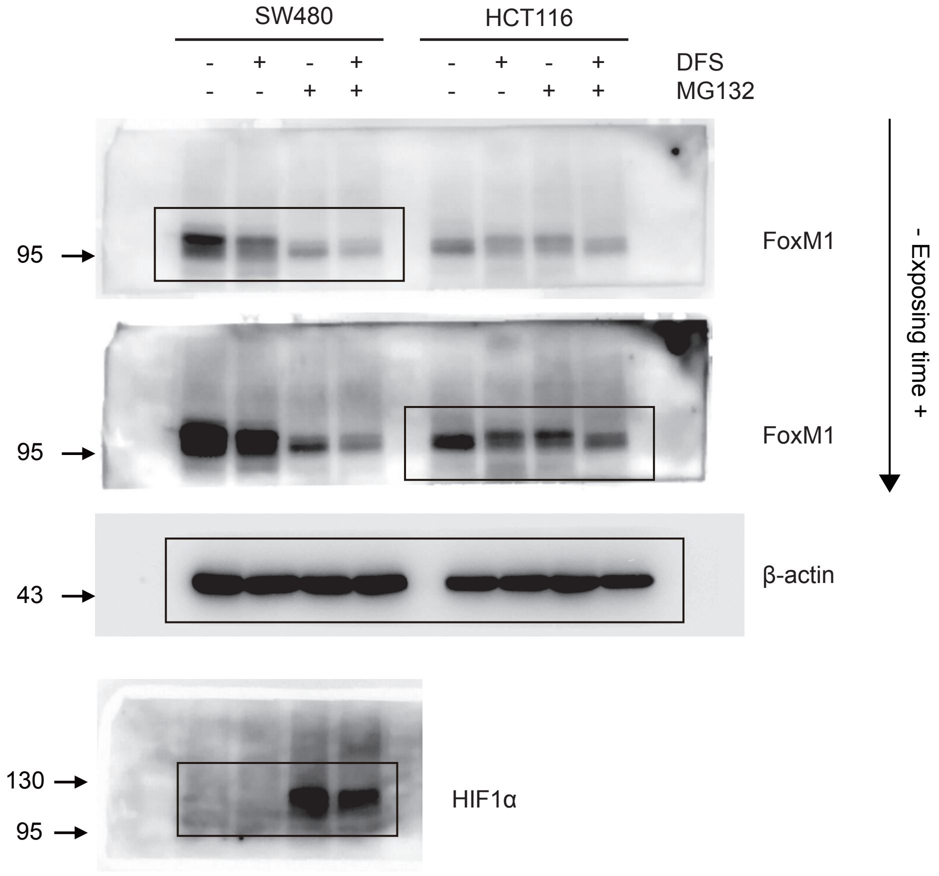

Figure S10i. original image of figure 4D.

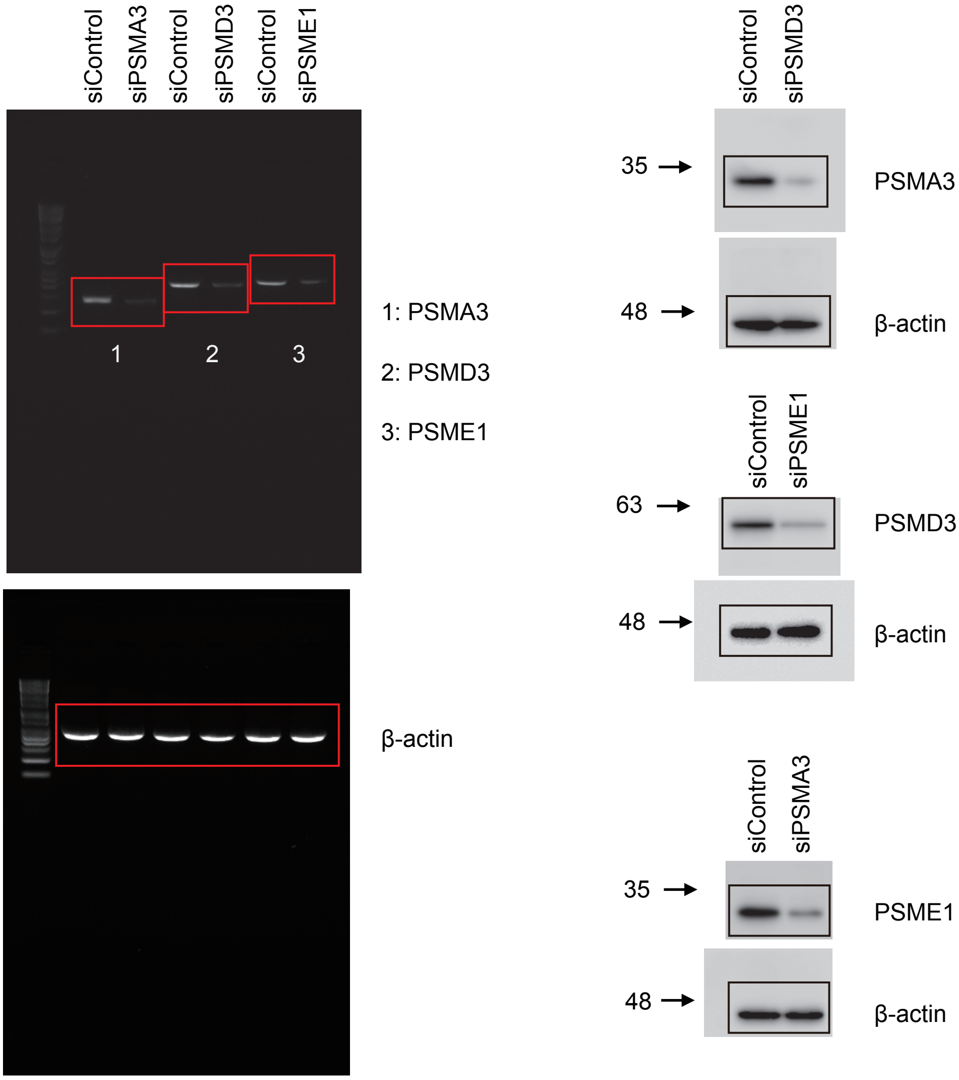

Figure S10j. original image of figure 4E.

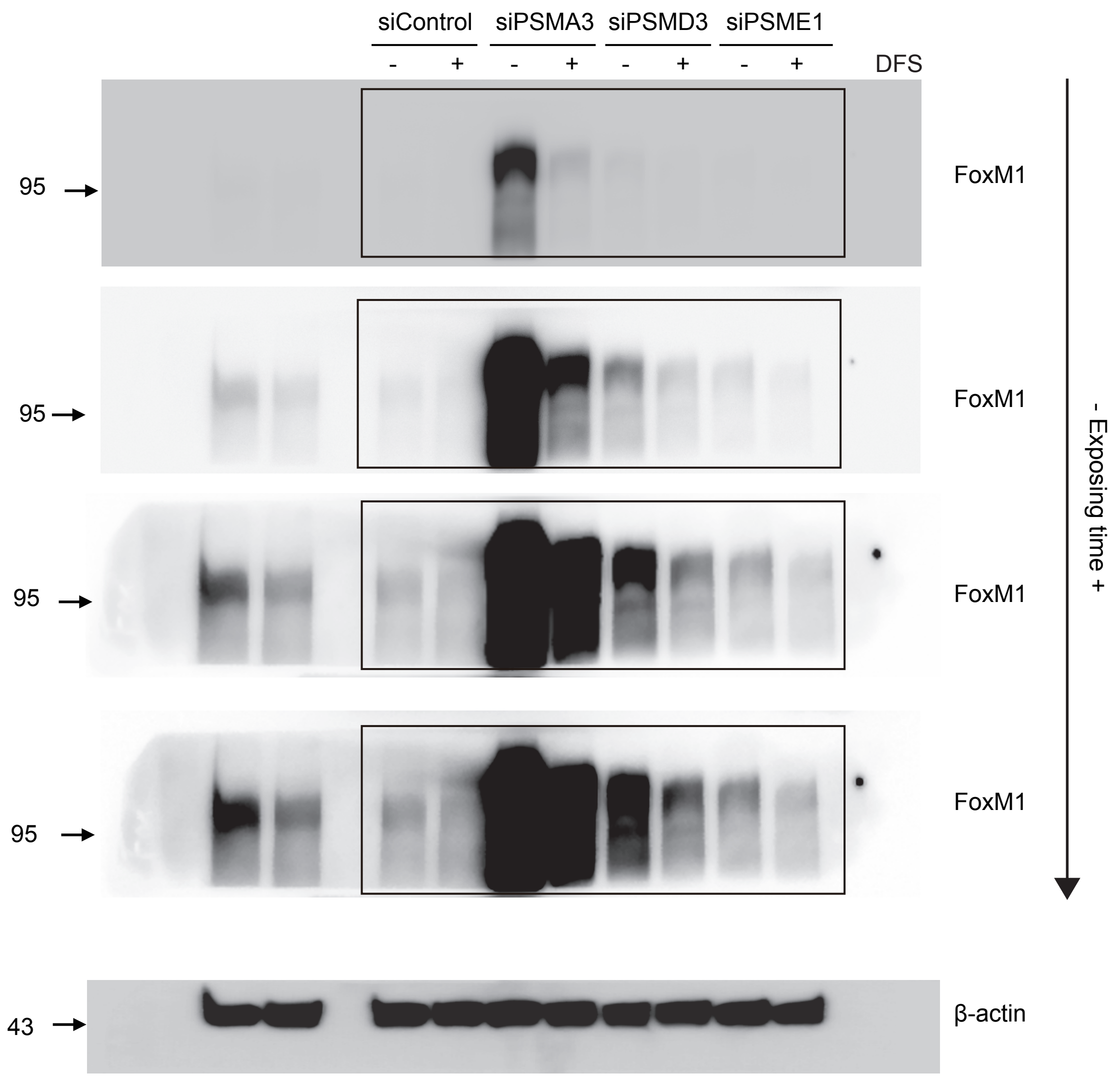

Figure S10k. original image of figure 4F.

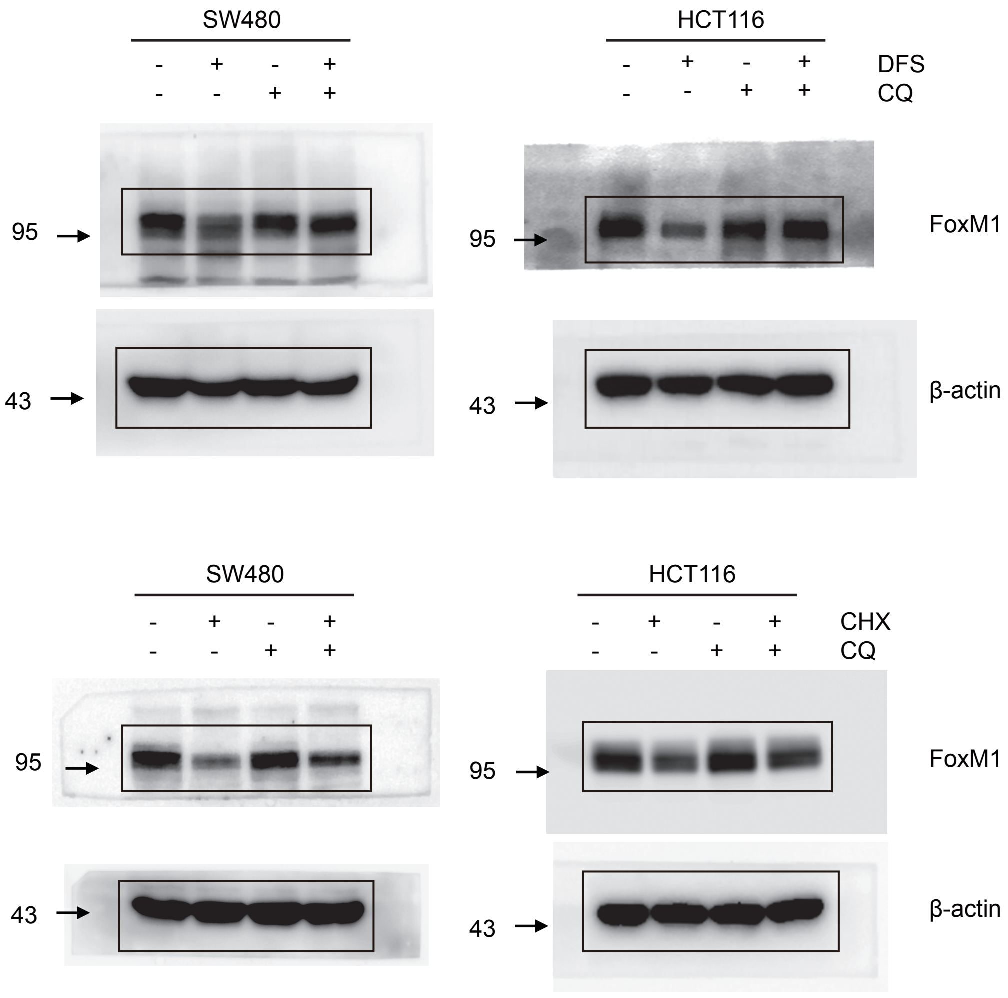

Figure S10l. original image of figure 5A.

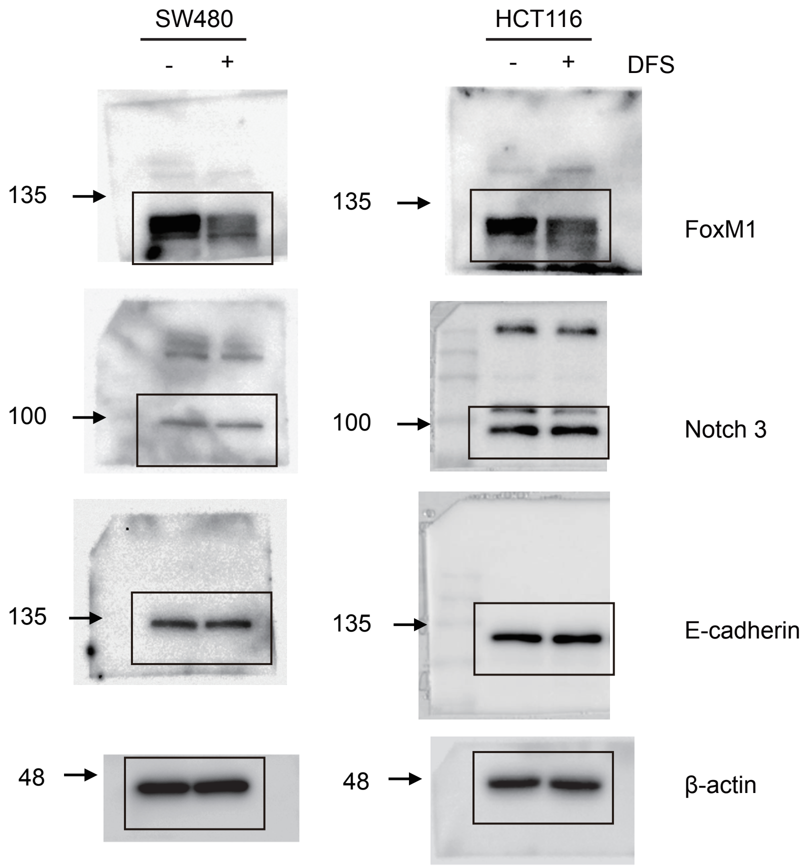

Figure S10m. original image of figure 5B.

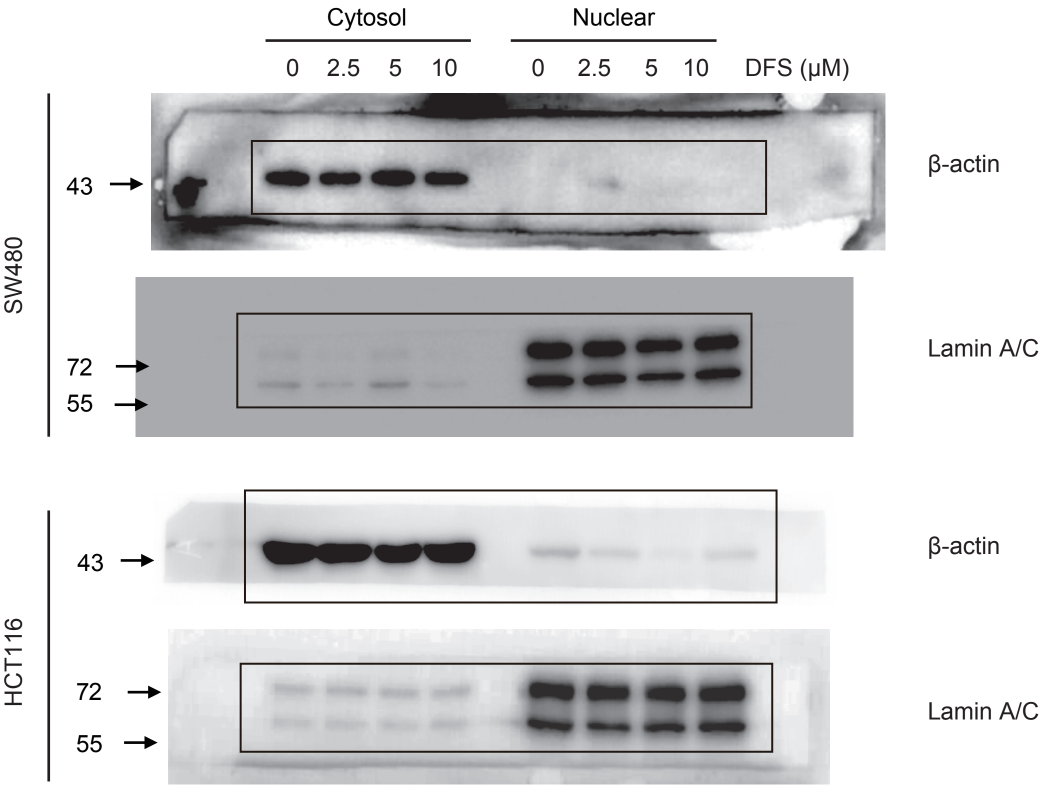

Figure S10n. original image of figure S7A.

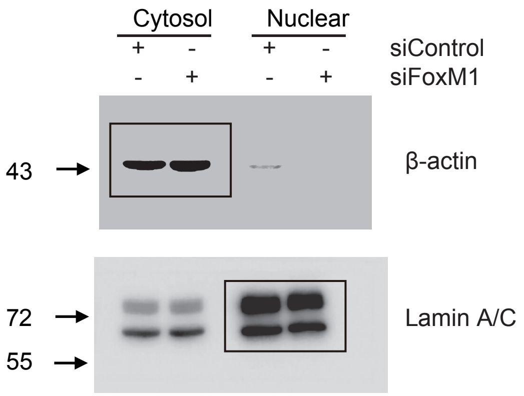

Figure S10o. original image of figure S7B.

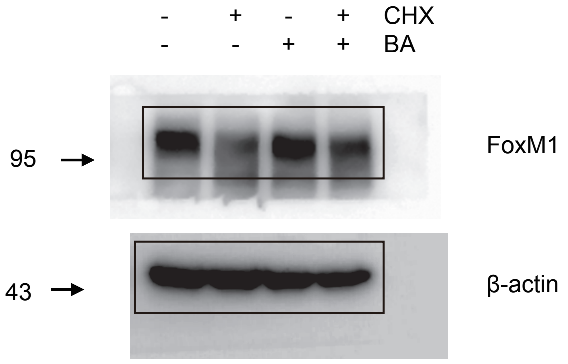

Figure S10p. original image of figure S8A.

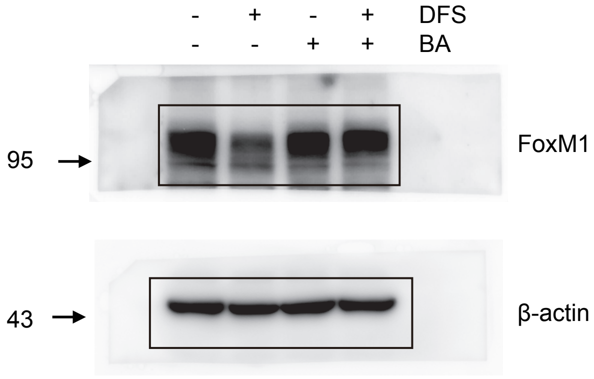

Figure S10q. original image of figure S8B.
